# Dynamic graph neural networks for UAV-based group activity recognition in structured team sports

**DOI:** 10.3389/fnbot.2025.1631998

**Published:** 2025-09-08

**Authors:** Ishrat Zahra, Yanfeng Wu, Haifa F. Alhasson, Shuaa S. Alharbi, Hanan Aljuaid, Ahmad Jalal, Hui Liu

**Affiliations:** ^1^Guodian Nanjing Automation Co., Ltd., Nanjing, China; ^2^Department of Computer Science, Air University, Islamabad, Pakistan; ^3^Department of Information Technology, College of Computer, Qassim University, Buraydah, Saudi Arabia; ^4^Computer Sciences Department, College of Computer and Information Sciences, Princess Nourah bint Abdulrahman University (PNU), Riyadh, Saudi Arabia; ^5^Department of Computer Science and Engineering, College of Informatics, Korea University, Seoul, Republic of Korea; ^6^School of Future Technology, Nanjing University of Information Science and Technology, Nanjing, China; ^7^Cognitive Systems Lab, University of Bremen, Bremen, Germany

**Keywords:** unmanned aerial vehicles, neural network models, machine learning, body pose, group action recognition, feature extraction, deep learning unmanned aerial vehicles, deep learning

## Abstract

**Introduction:**

Understanding group actions in real-world settings is essential for the advancement of applications in surveillance, robotics, and autonomous systems. Group activity recognition, particularly in sports scenarios, presents unique challenges due to dynamic interactions, occlusions, and varying viewpoints. To address these challenges, we develop a deep learning system that recognizes multi-person behaviors by integrating appearance-based features (HOG, LBP, SIFT), skeletal data (MediaPipe, MOCON), and motion features. Our approach employs a Dynamic Graph Neural Network (DGNN) and Bi-LSTM architecture, enabling robust recognition of group activities in diverse and dynamic environments. To further validate our framework’s adaptability, we include evaluations on Volleyball and SoccerTrack UAV-recorded datasets, which offer unique perspectives and challenges.

**Method:**

Our framework integrates YOLOv11 for object detection and SORT for tracking to extract multi-modal features—including HOG, LBP, SIFT, skeletal data (MediaPipe), and motion context (MOCON). These features are optimized using genetic algorithms and fused within a Dynamic Graph Neural Network (DGNN), which models players as nodes in a spatio-temporal graph, effectively capturing both spatial formations and temporal dynamics.

**Results:**

We evaluated our framework on three datasets: a volleyball dataset, SoccerTrack UAV-based soccer dataset, and NBA basketball dataset. Our system achieved 94.5% accuracy on the volleyball dataset (mAP: 94.2%, MPCA: 93.8%) with an inference time of 0.18 s per frame. On the SoccerTrack UAV dataset, accuracy was 91.8% (mAP: 91.5%, MPCA: 90.5%) with 0.20 s inference, and on the NBA basketball dataset, it was 91.1% (mAP: 90.8%, MPCA: 89.8%) with the same 0.20 s per frame. These results highlight our framework’s high performance and efficient computational efficiency across various sports and perspectives.

**Discussion:**

Our approach demonstrates robust performance in recognizing multi-person actions across diverse conditions, highlighting its adaptability to both conventional and UAV-based video sources.

## 1 Introduction

Group activity recognition is a challenging task in computer vision that aims to understand collaborative behaviors involving multiple individuals interacting with each other. Unlike individual action recognition, group activity recognition requires modeling both the actions of each individual and the complex interactions among participants (Wang and Mohamed, 2023; Zhang Y. et al., 2023; Zhang L. et al., 2023). This task is particularly challenging in sports settings, where dynamic movements, occlusions, varying viewpoints, and rapid transitions between activities complicate the recognition process. Traditional approaches have typically focused on either appearance-based feature extracted from RGB frames or skeletal representations of human poses (Yan R. et al., 2018; Yan S. et al., 2018; Askari et al., 2023) with each approach presenting its set of limitations (Wang and Yan, 2023). RGB-based methods often struggle with environmental variations, occlusions, and computationally intensive processing, while skeletal approaches frequently require meticulous pose annotation (Zhang Y. et al., 2023; Zhang L. et al., 2023) and sophisticated interaction modeling but may miss important visual cues present in full-frame data (Brdiczka et al., 2009). Recent advancements in deep learning have enabled more sophisticated approaches to group activity recognition (Tamura, 2024), particularly in sports analytics (Direkoğlu and O'Connor, 2012).

Transformer-based models have proven effective at understanding how participants relate to each other over long distances, while attention mechanisms (Li et al., 2021) highlight the most important interactions during key moments. However, these methods often require substantial computational resources and large annotated datasets, which may not be readily available in all contexts. Also, creating effective multi-modal frameworks (Brdiczka et al., 2009; Florea and Mihailescu, 2020) involves major difficulties, such as matching features, finding the best ways to combine data, and ensuring the system runs efficiently. Despite these challenges, the potential benefits of synthesizing both appearance- and structure-based information make multi-modal approaches (Li et al., 2009) particularly valuable for tasks of recognizing complex group activities that require understanding both individual elements and their relationships within a larger context (Beenhakker et al., 2020).

In this paper, we address these challenges by developing a novel multi-modal framework that synergistically combines both appearance-based and skeletal features for recognizing group activities in volleyball. Our approach leverages the complementary strengths of different feature types while mitigating their individual weaknesses. By combining strong full-body descriptions that show how players look and their body shapes with detailed features that represent how they move, our system gets a better overall picture of group activities. We also improve our method by using a Dynamic Graph Neural Network (DGNN) and Bi-LSTM that represents how players are positioned and move over time, helping to identify the teamwork patterns needed for correctly recognizing group activities. This multi-modal approach significantly improves recognition accuracy while maintaining computational efficiency, addressing a key limitation of many existing systems.

The main contributions of our work are:

- A novel multi-modal framework that effectively integrates appearance-based features (HOG, LBP, SIFT) with skeletal features (MediaPipe, MOCON) to provide a more robust representation of volleyball activities than modality alone.
- A hierarchical feature extraction pipeline that captures information at multiple levels of granularity, from individual player morphology to team-wide spatial arrangements and temporal dynamics.
- A computationally efficient approach to feature optimization and fusion that selectively reduces dimensionality while preserving discriminative information, making real-time processing more feasible.
- A Dynamic Graph Neural Network and Bi-LSTM architecture specifically designed for sports activity recognition that explicitly models the spatial and temporal relationships between players, capturing the collaborative nature of team sports.

## 2 Literature review

Group activity recognition has witnessed significant progress recently, as researchers have proposed various methodologies to address the challenges of understanding collective behaviors (Wang and Mohamed, 2023). Combining methods that look at how people look, their body movements, and their relationships has created better systems for recognizing group activities, like those seen in team sports such as volleyball (Ibrahim et al., 2016). This section reviews key works in the field, focusing on three primary areas: single-person activity recognition, multi-person activity recognition, and group activity recognition.

### 2.1 Single-person activity recognition

Researchers have extensively studied single-person activity recognition, focusing on both appearance and skeletal data (Shi et al., 2019) introduced Spatial Temporal Graph Convolutional Networks (ST-GCN), a model that uses human skeletons as graphs (Li et al., 2017) for action recognition. Their approach has been foundational in leveraging skeletal data for activity understanding. Another significant contribution is Chen et al. (2017), which reviewed depth and inertial sensor fusion methods, presenting various fusion techniques that have shown promise in improving recognition performance. The work of Wang and Yan (2023) on I3D models represents a pioneering approach for RGB-based action recognition, which has had a profound influence on the use of visual data in activity recognition. The NTU RGB + D 120 dataset created by Liu et al. (2020) offered a big standard for testing skeletal recognition methods, helping to create stronger models (Shi et al., 2019) built on previous skeletal methods by adding Directed Graph Neural Networks (DGNN), which tracked how information moves in skeletal structures, greatly enhancing performance in recognizing actions based on skeletons.

Recent advances in UAV-based human activity recognition have demonstrated the effectiveness of both CNN and transformer-based architectures for aerial surveillance applications, presenting unique challenges that distinguish aerial analysis from ground-based approaches. Maheriya et al. (2024) present a comprehensive CNN-based approach tailored for diverse aerial conditions, demonstrating that specialized CNN configurations can maintain recognition accuracy despite inherent challenges of aerial data collection such as varying scale, perspective distortion, and atmospheric interference. Their work achieved robust performance across varying environmental conditions including different altitudes, weather conditions, and lighting scenarios, highlighting the importance of environmental adaptation in UAV-based recognition systems. Yadav et al. (2023) proposed a novel Sparse Weighted Temporal Attention (SWTA) module for drone-camera based activity recognition that utilizes sparsely sampled video frames for obtaining global weighted temporal attention, demonstrating significant improvements in UAV-based human activity recognition by combining CNN feature extraction with temporal attention mechanisms to handle the complex poses and environmental scenarios inherent in aerial surveillance applications.

These UAV-specific developments complement our multi-modal DGNN approach by highlighting the importance of robust feature integration and adaptive processing for aerial applications, where individual modalities may be compromised by altitude, weather, or perspective variations that our graph-based relationship modeling can effectively address.

### 2.2 Multi- person activity recognition

In the context of multi-person activity recognition, several methods have emerged to capture the complex interactions between individuals within a group (Perez et al., 2022) proposed the Interaction Relational Network for mutual action recognition, which highlights the importance of modeling interactions between individuals. Their work laid the groundwork for the study of multi-person interactions, particularly in collaborative environments. Similarly, Bagautdinov et al. (2016) introduced the person–person interaction model, a hierarchical approach that aims to improve multi-person activity understanding by considering both individual and group-level interactions. Other studies, like Chang et al. (2015), investigated multi-instance contrastive learning for recognizing group activities, using a multi-modal approach that highlighted how contrasting learning signals can enhance recognition. The two-level attention-based interaction model from Lu et al. (2018) built on these concepts by using skeleton data to identify interactions, showing how useful multi-modal data (Xie et al., 2025) can be in understanding complex human behaviors. The two-level attention-based interaction model from Lu et al. (2018) improved these ideas by using skeleton data to recognize interactions, highlighting how helpful multi-modal data can be in understanding complex human behaviors.

### 2.3 Group activity recognition

Understanding group interactions and modeling collective actions to account for multiple participants has been the focus of group activity recognition (Ibrahim et al., 2016) proposed a hierarchical deep temporal model, which processes individual actions before integrating them to recognize group activities, with a particular emphasis on volleyball activity recognition. Building on this work, Gavrilyuk et al. (2020) introduced Actor-Transformers, which employ transformer-based architectures to model group activities more efficiently. Their model benefits from the self-attention mechanism to capture player interactions, but faces challenges in real-time applications due to high computational costs. In a similar vein, Wu et al. (2019) explored learning actor relation graphs for group activity recognition, offering an approach that emphasizes the relationships between actors in a group. This method shares many similarities with the graph-based approaches employed in modern multi-modal frameworks for group activity recognition (Biswas and Gall, 2018) suggested a network that looks at players’ movements over time on different scales, providing an additional way to recognize group activities by understanding how space and time are connected. Abbas et al. (2024) developed unmanned aerial vehicles for human detection and recognition using neural-network models, achieving robust performance in UAV applications and demonstrating that deep learning architectures can effectively handle unique challenges of aerial perspectives while maintaining processing capabilities suitable for real-time surveillance applications. The integration of edge computing with UAV-based recognition has gained significant attention due to power and processing constraints in aerial platforms. Kapoor et al. (2024) conducted a comprehensive survey on human action recognition in aerial videos, analyzing various CNN-based approaches and their effectiveness across different UAV deployment scenarios, demonstrating how deep learning architectures can adapt to scale variations and perspective changes inherent in aerial recognition applications. Bany Abdelnabi and Rabadi (2024) created a state-of-the-art review of human detection from unmanned aerial vehicles’ images for search and rescue missions, achieving comprehensive analysis of lightweight architectures specifically optimized for UAV deployment, proving that intelligent architecture design can balance recognition performance with computational constraints for practical aerial surveillance applications.

The work of Li et al. (2021) contributed to an efficient transformer model for group activity recognition by employing dense local attention mechanisms. This model addressed the challenge of maintaining computational efficiency while capturing the dynamics of group activities. Additionally, Ehsanpour et al. (2020) introduced a framework for joint learning for social groups, individual actions, and subgroup activities, recognizing the hierarchical nature of group activities in videos. Their approach, though successful in modeling group activity, faces limitations in handling fast-paced sports environments where the composition of groups rapidly changes. Further expanding on this area, Beenhakker et al. (2020) introduced the concept of error impact in individual action classification when modeling group interactions in volleyball, particularly when errors in single-person action recognition affect overall group activity modeling. Their work emphasizes the need for robust classification models for action recognition, which can directly influence group activity recognition accuracy. Finally, Li and Chuah (2017) in their SBGAR approach, proposed semantics-based group activity recognition, achieving significant results on the Volleyball dataset (Ibrahim et al., 2016), which integrates semantic information for enhanced group activity classification. This method offers a practical solution for group activity recognition by focusing on semantic relationships (Tang et al., 2018) and leveraging multiple feature representations for better accuracy.

### 2.4 Architectural trade-offs: CNN vs. transformer vs. graph-based approaches

The choice of neural architecture fundamentally impacts how group activity recognition systems process spatial relationships and temporal dependencies in multi-person scenarios. CNN-based approaches excel at extracting hierarchical spatial features through local receptive fields, with methods like Ibrahim et al. (2016) achieving 81.9% accuracy through efficient parameter sharing and translation equivariance that makes them suitable for real-time applications. However, CNNs face limitations in capturing long-range spatial dependencies between distant players, requiring deep networks or additional mechanisms to model global context effectively. Vision Transformers represent a paradigm shift toward global attention mechanisms that can capture long-range dependencies between any pair of players regardless of spatial distance. Maheriya et al. (2025) demonstrated that ViTs can effectively recognize athletic activities across varied sporting domains by leveraging self-attention mechanisms that dynamically weight player importance based on activity context. Gavrilyuk et al. (2020) achieved 89.3% accuracy through Actor-Transformers that utilize attention to model player interactions without explicit spatial constraints, though transformers require quadratic computational complexity and substantially higher memory requirements compared to CNN approaches.

Graph-based approaches, including our proposed DGNN framework, offer a middle ground that explicitly models player relationships through structured representations while maintaining computational tractability. Unlike transformers that compute attention between all possible player pairs (Li et al., 2022), graph networks focus on meaningful relationships defined by domain knowledge or learned constraints. Wu et al. (2019) demonstrated that Actor Relation Graphs achieve 89.5% accuracy by explicitly modeling spatial relationships, showing that structured relationship modeling can compete with attention-based approaches (Huan et al., 2023) while providing greater interpretability. Our DGNN approach captures both local player interactions and global team coordination through rule-aware graph construction that incorporates sport-specific constraints, enabling more efficient processing than transformers while avoiding the local limitations of CNN architectures. Performance analysis reveals that hybrid approaches combining multiple paradigms, like our framework achieving 94.5% accuracy, can outperform pure transformer methods while maintaining computational efficiency suitable for practical deployment, suggesting that architectural diversity provides optimal solutions for complex group activity recognition tasks.

### 2.5 Superiority of our approach over other methods

Previous approaches to group activity recognition have been constrained by single-modality limitations or high computational demands. Transformer-based methods (Li et al., 2021; Gavrilyuk et al., 2020) capture interactions effectively, but remain computationally prohibitive, while graph-based (Yan R. et al., 2018; Yan S. et al., 2018) and skeletal approaches (Askari et al., 2023; Zappardino et al., 2021) often miss crucial contextual information. Our multi-modal framework overcomes these limitations by integrating appearance-based features with skeletal and motion representations, employing genetic algorithm optimization to reduce dimensionality while preserving discriminative information, achieving superior performance with lower computational requirements.

Our methodology improves upon previous models for recognizing group activities by integrating appearance-based features (HOG, LBP, SIFT) with skeletal features (MediaPipe), and motion cues (MOCON). This multi-modal approach provides a more comprehensive understanding of volleyball activities, addressing the limitations of single-modality systems. We employ a genetic algorithm for feature optimization, reducing dimensionality and computational overhead compared to transformer-based models, which are computationally expensive. Additionally, our Dynamic Graph Neural Network (DGNN) effectively captures spatio-temporal relationships between players, overcoming the inefficiencies of prior methods. This combination enhances recognition accuracy (94.5% on the volleyball dataset, 91.8% on the SoccerTrack UAV dataset, and 91.1% on NBA dataset) while maintaining suitability for sports group activity recognition applications.

## 3 Materials and method

### 3.1 System methodology

**Figure 1:**
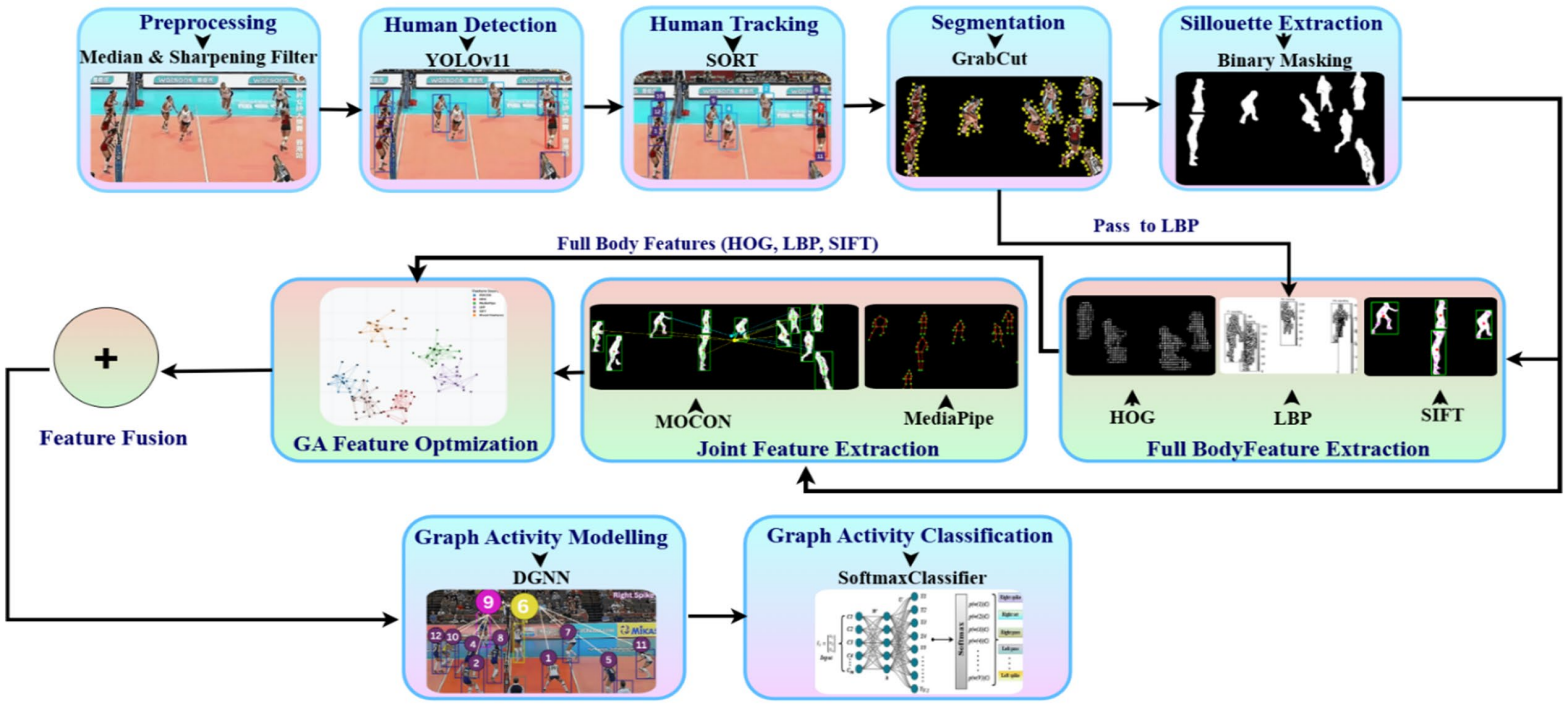
Architectural framework for group activity recognition showing the complete processing pipeline from preprocessing through feature extraction, optimization, graph modeling with DGNN, and final classification.

Our methodology tackles the complex challenge of group activity detection through a multi-stage approach that processes visual data from raw frames to meaningful behavioral patterns. The system employs sophisticated filtering techniques, advanced human detection, precise feature extraction, and graph-based relational modeling to accurately classify group activities. As shown in Figure 1, each part of the process is made to gradually improve the information taken from the video frames, leading to a strong classification system.

### 3.2 Pre-processing

We implement a dual-filtering approach consisting of median filtering for noise reduction, followed by edge-preserving sharpening to accentuate significant visual features in volleyball sequences. Initially, input frames undergo a nonlinear median filtering operation to suppress impulse noise while preserving important edge information. For each pixel coordinate in the input frame *I*, the median filter computes (as mentioned in Equation 1):


(1) $$I_{\mathrm{med}}(x,y)=\text{median}\{I(x+i,y+j)|-\frac{k-1}{2}\leq i,j\leq \frac{k-1}{2}\}$$

where *k* represents the kernel size (empirically set to 3 in our implementation), and $I_{\mathrm{med}}$ is the resulting filtered image. The median operation effectively eliminates outlier pixel values by replacing each pixel with the median value from its neighborhood, preserving critical structural information while removing noise artifacts that could impede subsequent analysis.

**Figure 2:**
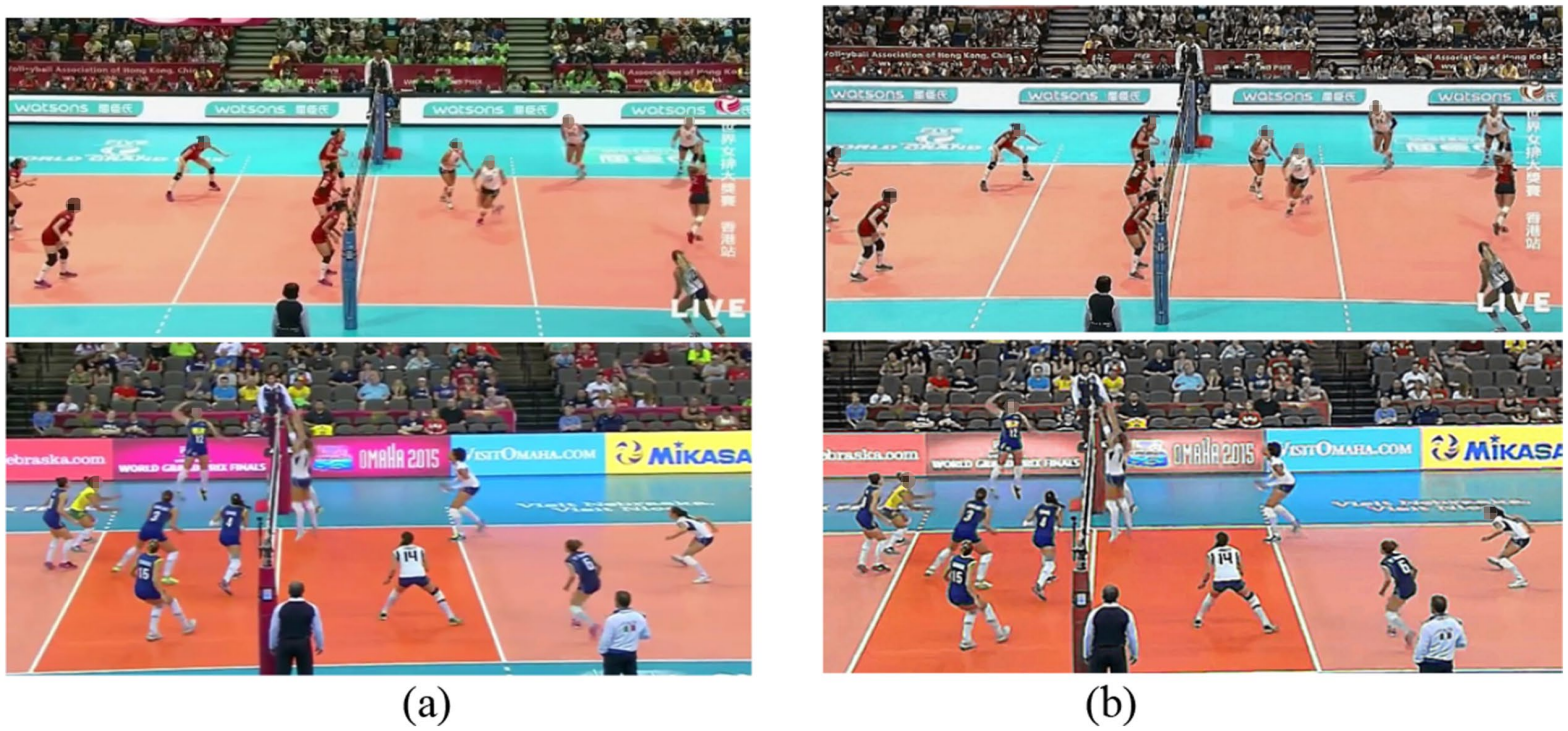
Comparison between **(a)** original input frames and **(b)** corresponding enhanced images after preprocessing.

Following noise reduction, we apply a spatial sharpening filter to enhance edge definition and detail visibility. This is accomplished through a convolution operation with a specialized kernel designed to amplify local contrast (as mentioned in Equation 2):


(2) $$I_{\text{sharp}}(x,y)=\sum_{i=-1}^{1}\sum_{j=-1}^{1}K(i,j)\cdot I_{\mathrm{med}}(x+i,y+j)$$

where the kernel K(i,j) acts as a filter, it is applied to each pixel by evaluating its surrounding neighborhood. This convolution enhances edges and details in the image by amplifying the differences between neighboring pixels, resulting in a sharper, clearer image (as illustrated in Figure 2, which compares raw input frames with their preprocessed frames, demonstrating significant improvement in visual clarity and edge definition critical for subsequent feature extraction).

### 3.3 Human detection

For human detection, we implement state-of-the-art YOLOv11 architecture, which represents a significant advancement over previous object detection frameworks (Khanam and Hussain, 2024). We selected YOLOv11 after extensive comparative analysis against other detection frameworks (RCNN variants, SSD, and earlier YOLO versions), as it demonstrates superior performance metrics for human detection in sports contexts, with a 7.8% improvement (see Section 4.4.1) in mean Average Precision (mAP) and 43% faster inference time compared to YOLOv10. YOLOv11 employs a dense prediction architecture that optimizes objectness, class probability, and localization in a unified framework. Each grid cell predicts several bounding boxes, and the confidence for each detection is calculated by adding the objectness score and the class probability using sigmoid activations. Specifically, the final human detection confidence score for a bounding box is given in Equation 3:


(3) $${\hat{C}}_{\mathrm{ij}}=\sigma ({\hat{p}}_{\mathrm{obj}}^{\mathrm{ij}})\cdot \sigma (\max_{c\in C}{\hat{p}}_{c}^{\mathrm{ij}}),\;\;C=\{\text{Human}\}$$

where ${\hat{p}}_{\mathrm{obj}}^{\mathrm{ij}}$ denotes the predicted objectness logit, ${\hat{p}}_{c}^{\mathrm{ij}}$ the predicted class score for the class c , and σ(·) is the sigmoid activation function. This formulation ensures that high confidence is attributed only to boxes that are both likely to contain an object and classified as “Human.”

The detection architecture employs a composite loss function that balances three critical components, which is mathematically expressed in Equation 4:


(4) $${ℒ}_{\text{YOLO}}=\lambda_{\mathrm{loc}}{ℒ}_{\mathrm{loc}}+\lambda_{\text{conf}}{ℒ}_{\text{conf}}+\lambda_{\text{class}}{ℒ}_{\text{class}}\;$$

where ${ℒ}_{\mathrm{loc}}$ penalizes bounding box coordinate errors, ${ℒ}_{\text{conf}}$ addresses confidence score accuracy, and ${ℒ}_{\text{class}}$ focuses on classification accuracy. The λ terms represent weighting parameters that control the relative importance of each component during training.

**Figure 3:**
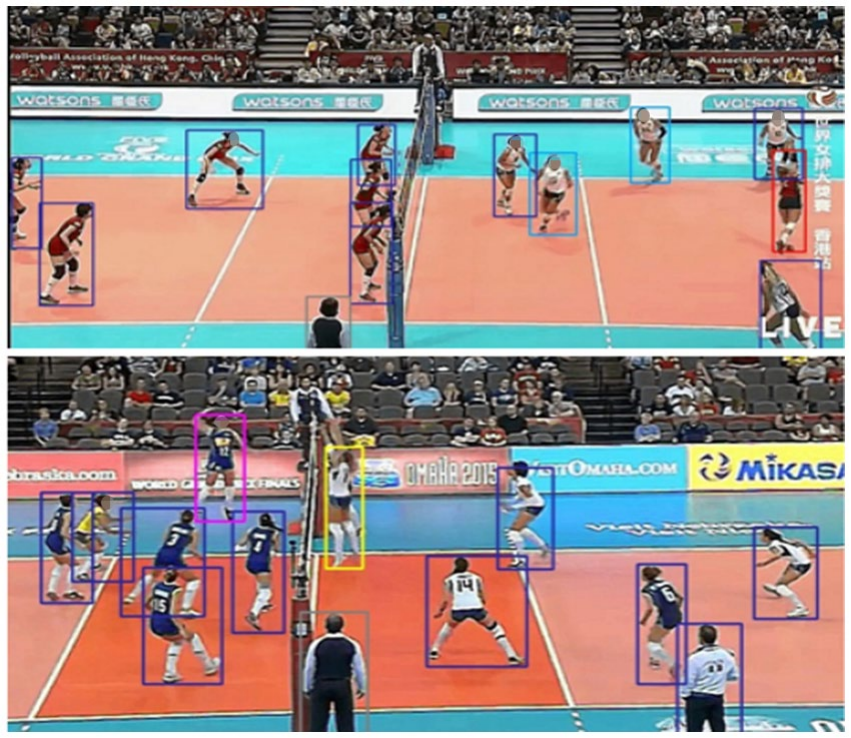
YOLOv11 detection results showing accurate and reliable player localization.

This formulation enables YOLOv11 to accurately detect volleyball players even under challenging conditions, including occlusions, varied postures, and rapid movements that are common in volleyball sequences, as illustrated in Figure 3.

### 3.4 Human tracking

**Figure 4:**
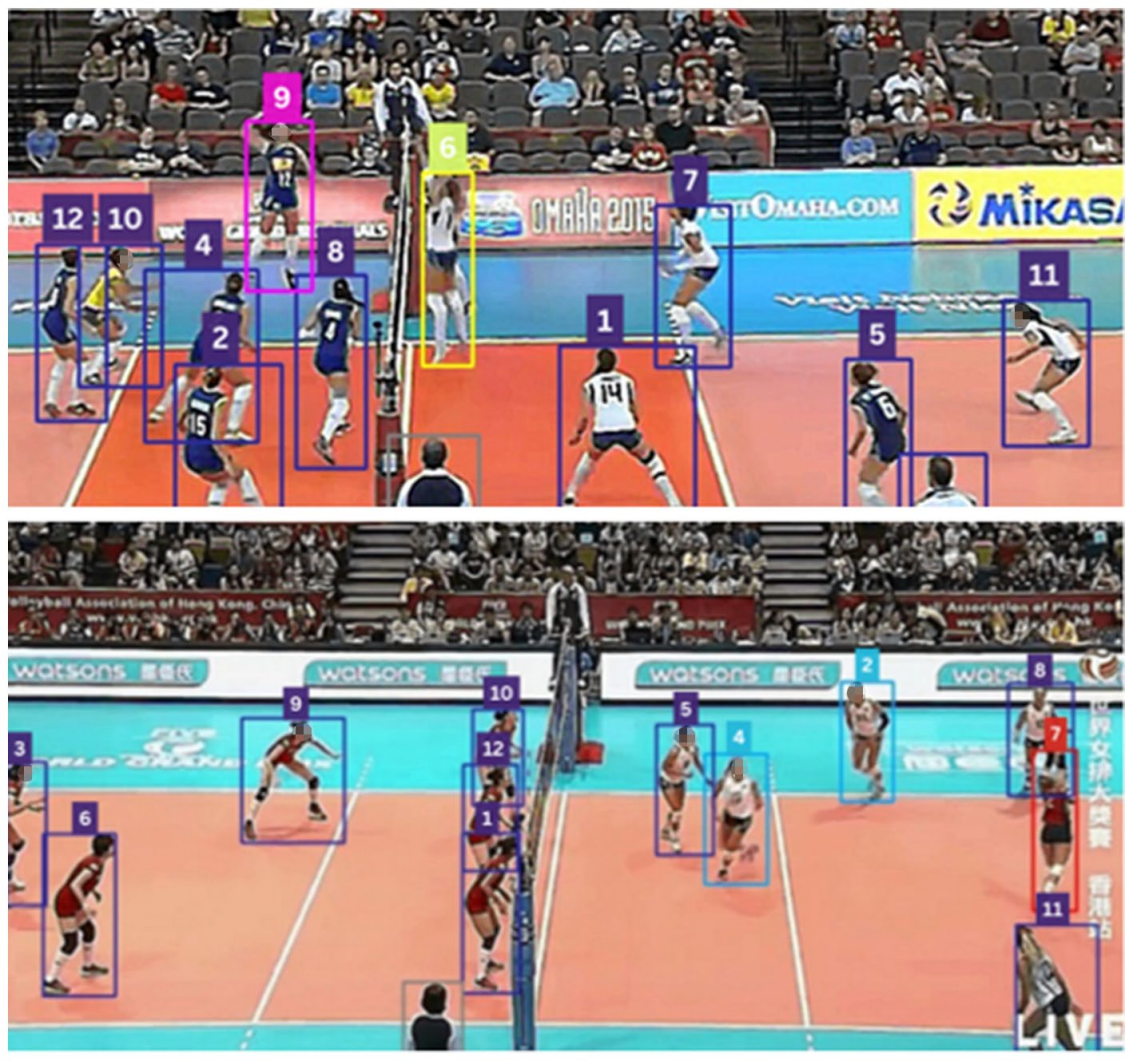
SORT results precisely tracking multiple players on both sides of the court.

To maintain a consistent player identity across sequential frames, we integrate the Simple Online and Realtime Tracking (SORT) algorithm (Cao et al., 2023). SORT effectively addresses the temporal association problem by assigning unique identifiers to detected players and maintaining their continuity throughout the volleyball sequence. This tracking mechanism employs Kalman filtering with a constant velocity motion model and the Hungarian algorithm for data association. For each detected player, the state vector $x={\left[u,v,s,r,\dot{u},\dot{v},\dot{s}\right]}^{T}$ is tracked, where (u,v) represents the bounding box center position, s is scale (area), r is the aspect ratio, and $\left(\dot{u},\dot{v},\dot{s}\right)$ are their respective velocities. The state prediction is governed by the linear dynamical system illustrated in Equation 5, which characterizes the temporal evolution of player states:


(5) $$x_{k}=Fx_{k-1}+w_{k}$$

where F is the state transition matrix and $w_{k}∼N(0,Q)$ represents process noise. The observation model relates the state vector to measurements $z_{k}$ is given in Equation 6:


(6) $$z_{k}={\mathrm{Hx}}_{k}+v_{k}$$

where H is the observation matrix and $v_{k}∼N(0,R)$ is measurement noise. Figure 4 illustrates the precise tracking performance of SORT, accurately following multiple players on both sides of the court.

### 3.5 Segmentation

For precise player segmentation, our methodology incorporates the GrabCut algorithm within the detected bounding boxes (Tang et al., 2013). GrabCut offers superior boundary delineation for human figures in volleyball contexts, particularly when players exhibit complex postures or partial occlusions. The GrabCut segmentation process operates on each detected player region defined by the bounding box. The algorithm models pixel distributions as Gaussian Mixture Models (GMMs) for foreground and background, as shown in Equation 7.


(7) $$p(z|\alpha ,k)=\sum_{k=1}^{K}\pi_{k,\alpha }N(z|\mu_{k,\alpha },\Sigma_{k,\alpha })\;$$

where z represents pixel color values, α∈{FG,BG} indicates foreground or background, $\pi_{k,\alpha }$ are mixture weights, and $\left(\mu_{k,\alpha },\Sigma_{k,\alpha }\right)$ are Gaussian parameters. The energy function for segmentation is given in Equation 8 as:


(8) E(α,k,θ,z)=U(α,k,θ,z)+V(α,z)

where U is the data term encouraging pixel assignment to its most likely GMM component, and V , as presented in Equation 9, is a smoothness term penalizing discontinuities between neighboring pixels:


(9) $$V(\alpha ,z)=\gamma \sum_{(p,q)\in N}\delta_{\alpha_{p}\neq \alpha_{q}}\exp(-\beta \|z_{p}-z_{q}\|2)$$

**Figure 5:**
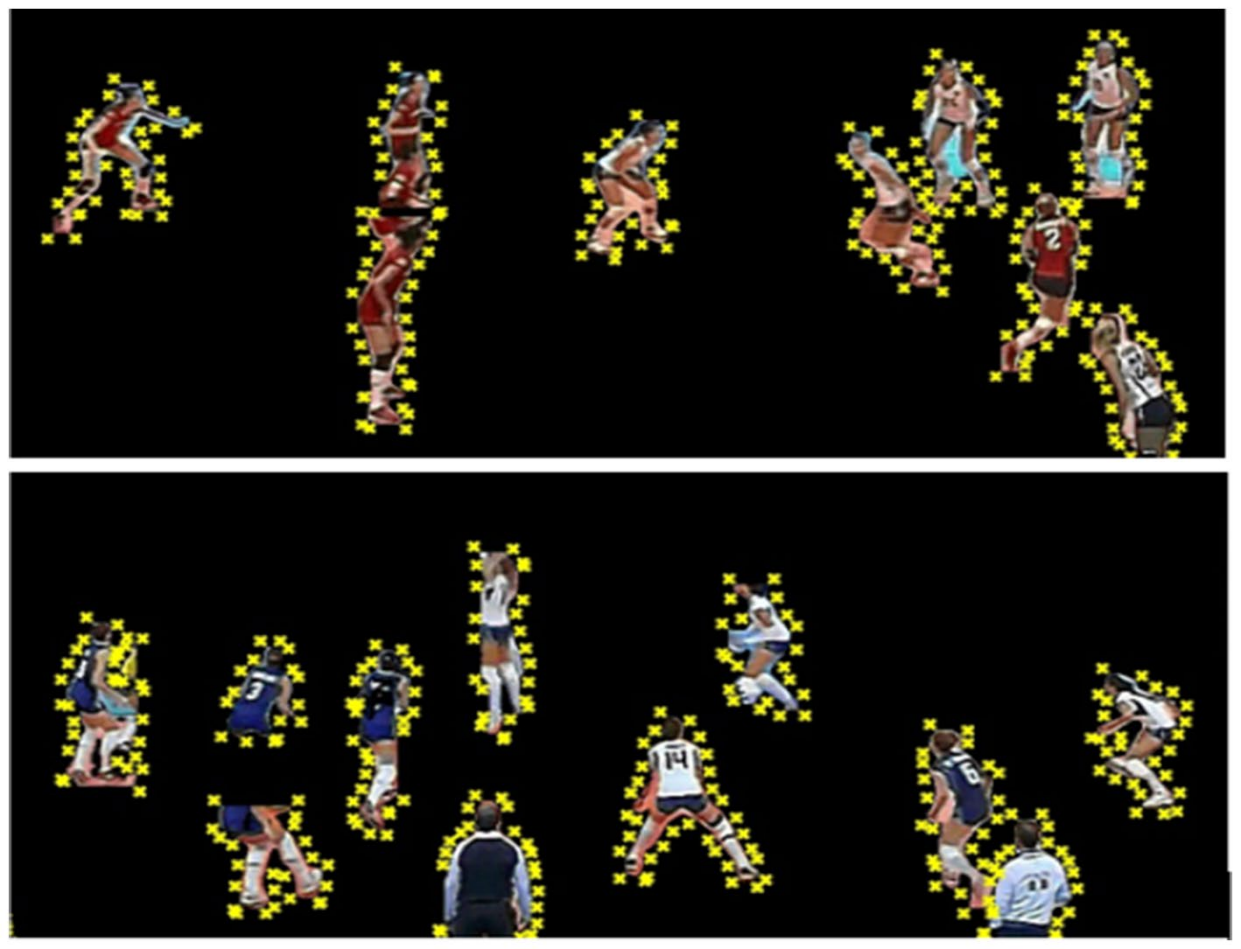
Multi-player segmentation results demonstrating the robust isolation of players from complex court backgrounds using the GrabCut algorithm.

The GrabCut algorithm iteratively estimates GMM parameters and minimizes the energy function using graph cuts, resulting in precise player segmentation. This approach achieves a significant improvement in boundary accuracy compared to alternative segmentation methods, as shown in Figure 5, providing high-quality inputs for subsequent feature extraction stages.

### 3.6 Silhouette extraction

**Figure 6:**
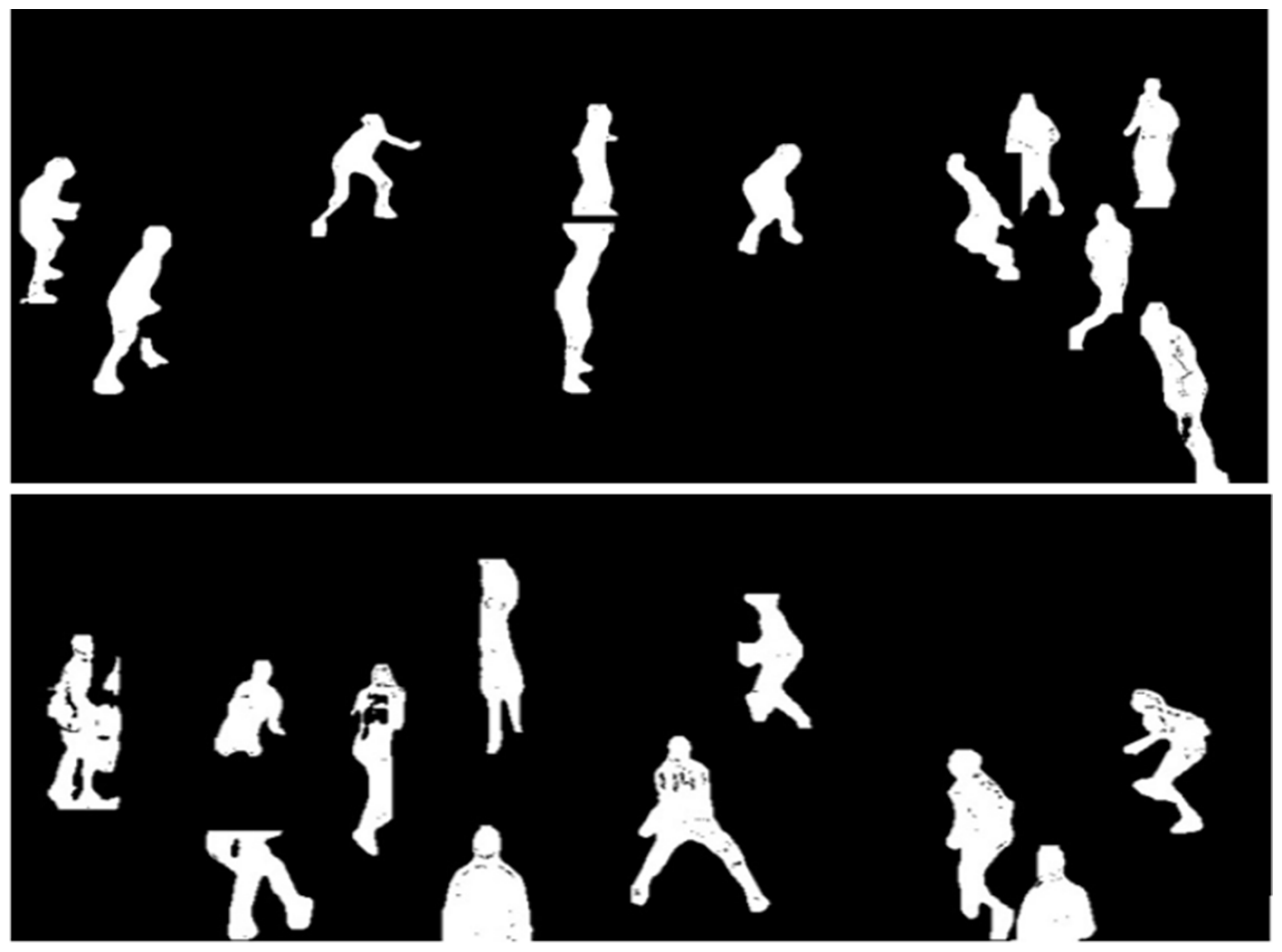
Extracted player silhouettes for quantitative activity analysis and motion pattern recognition.

To isolate player morphology, we convert the segmented players into binary silhouettes through thresholding. This process converts complex visual information into a simplified representation focusing solely on player shape, which is essential for subsequent pose and action analysis. The silhouette extraction process is given in Equation 10:


(10) $$S_{i}(x,y)=\{\begin{array}{cc} 1 & \text{if}\;G(I_{\mathrm{seg},i}(x,y))>\tau \\ 0 & \text{otherwise} \end{array}$$

where $I_{\mathrm{seg},i}$ is the segmented player region, G(·) is a grayscale conversion function, and τ is an adaptive threshold determined via Otsu’s method (as described in Equation 11):


(11) $$\begin{array}{l} \tau_{\text{Otsu}}=\arg\max_{\tau }\{\sigma_{B}^{2}(\tau )\}=\arg\max_{\tau }\\ \;\;\;\;\;\;\;\;\;\;\;\;\;\;\{w_{0}(\tau )w_{1}(\tau )[{\mu_{0}\left(\tau \right)-\mu_{1}\left(\tau \right)]}^{2}\} \end{array}$$

where $w_{0}$ and $w_{1}$ are the probabilities of the two classes separated by threshold *τ*, and $\mu_{0}$ and $\mu_{1}$ are the mean values of these classes. The resulting binary silhouettes provide a robust foundation for morphological analysis and feature extraction in the subsequent stages of our pipeline. It effectively captures the essential shape information while eliminating superfluous visual details and background noise, as demonstrated in Figure 6.

Our comprehensive human detection and segmentation framework ensures accurate player localization, consistent tracking, precise segmentation, and clean silhouette extraction, all critical prerequisites for the feature extraction and activity classification components that follow in our volleyball activity recognition system.

### 3.7 Full-body features

Building on human detection and segmentation, our methodology extracts full-body features using HOG, LBP, and SIFT to capture players’ visual characteristics. HOG helps identify the shape and structure of players to tell apart different volleyball positions, LBP makes sure textures are represented well, even with changing light, and SIFT finds key points that stay the same regardless of size or rotation to keep motion consistent. These complementary techniques enhance our framework’s ability to differentiate similar volleyball activities while maintaining computational efficiency.

#### 3.7.1 Histogram of oriented gradients (HOG)

The HOG descriptor accurately represents the shape and structure of volleyball players by measuring the direction and spread of gradients in specific areas of the players. This technique is particularly suitable for volleyball activity recognition as it excels at capturing the distinctive postures and contours associated with different volleyball actions such as spiking, blocking, and digging. For HOG feature extraction, we can formalize the process using a more detailed mathematical expression given in Equation 12:


(12) $$\begin{array}{l} F_{\mathrm{HOG}}(I)=\\ \;\;\{H_{i,j}=\sum_{p\in C_{i,j}}\omega (p)\cdot \delta (\theta (p)-b)\;\;i\in \{1,\ldots ,n_{c}\},j\in \{1,\ldots ,n_{c}\}\} \end{array}$$

where $H_{i,j}$ represents the histogram for the cell (i,j) , $C_{i,j}$ is the set of pixels in that cell, ω(p) is the gradient magnitude at pixel p , θ(p) is the gradient orientation, δ is the binning function that assigns gradient orientations to histogram bins b , and $n_{c}$ is the number of cells along each dimension of the image I . Each histogram aggregates gradient magnitudes within orientation bins as mentioned below in Equation 13:


(13) $$h_{i,j}[b]=\sum_{(x,y)\in {\text{cell}}_{i,j}}w(x,y)\cdot 1_{\theta (x,y)\in {\mathrm{bin}}_{b}}\;$$

where w(x,y) is the gradient magnitude at pixel (x,y) , θ(x,y) is the gradient orientation, and 1 is the indicator function.

**Figure 7:**
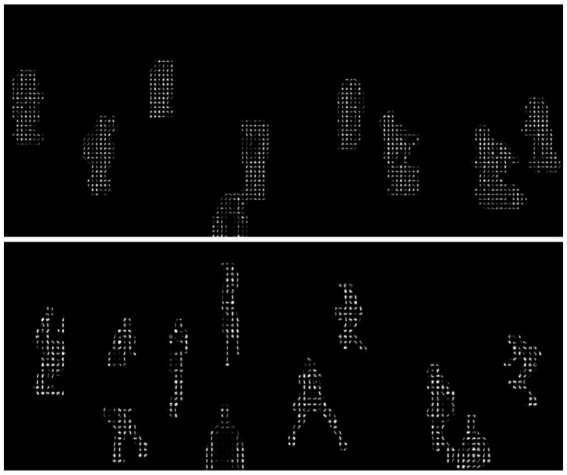
Histogram of Oriented Gradients (HOG) features highlighting player shape and motion cues.

HOG implementation is computationally efficient, using activity-specific configurations that adjust parameters based on the nature of the movement (Patel et al., 2020). For instance, more orientation bins are allocated for dynamic activities like spiking (12 orientations with 4 × 4 pixel cells), while simpler postures like waiting use fewer orientation bins (6 orientations with 16 × 16 pixel cells). This adaptive approach reduces computational overhead while maintaining discriminative power, as visualized in Figure 7.

#### 3.7.2 Local binary patterns (LBP)

To add to the structural details from HOG, we gather texture-based appearance features using LBP, which picks up small surface patterns and local brightness changes in the separate player areas (Truong et al., 2024). LBP offers robust performance under varying lighting conditions, making it ideal for the dynamic volleyball environment. The LBP feature extraction process is defined as in Equation 14:


(14) $$\mathrm{LB}P_{P,R}(x_{c},y_{c})=\sum_{p=0}^{P-1}s(g_{p}-g_{c})\cdot 2^{p}\;$$

where $\left(x_{c},y_{c}\right)$ is the center pixel, $g_{c}$ is its intensity, $g_{p}$ are the intensities of P equally spaced pixels on a circle of radius R , and s(x) is the step function given in Equation 15:


(15) $$s(x)=\{\begin{array}{cc} 1 & \text{if}\;x\geq 0\\ 0 & \text{otherwise} \end{array}$$

**Figure 8:**
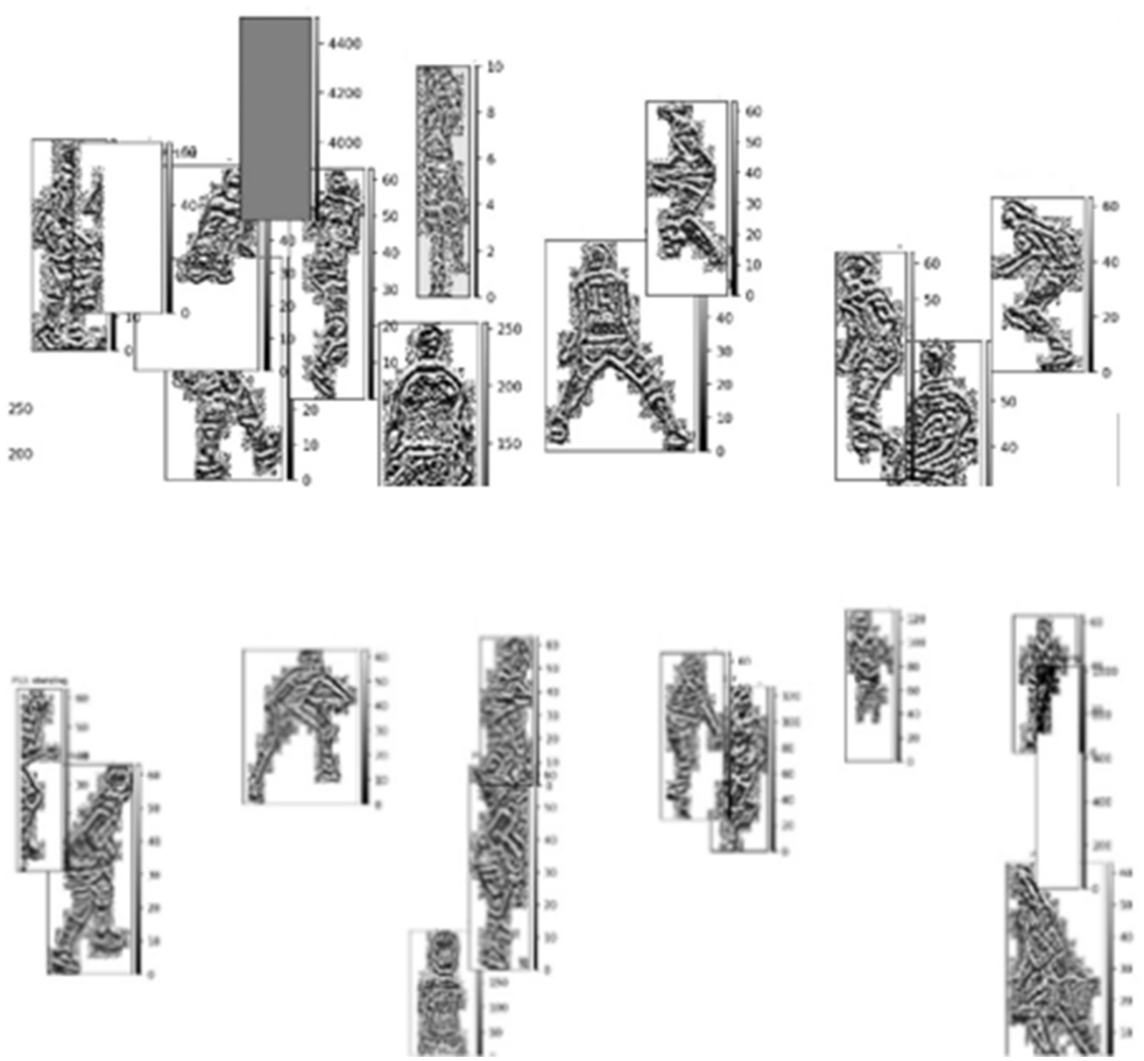
Local Binary Pattern (LBP) features highlighting the fine-grained texture details of the players for enhanced pattern analysis.

We utilize activity-specific configurations for LBP parameters, varying the sampling points P and radius R based on the action class, with uniform patterns for stationary activities and rotation-invariant patterns for dynamic movements. This tailored approach ensures optimal feature extraction while maintaining computational efficiency. LBP is exceptionally lightweight, with the computational complexity of O(P·n) , where P is the number of sampling points and n is the number of pixels. The operation involves simple pixel comparison operations without floating-point arithmetic, making it one of the most computationally efficient texture descriptors available, as shown in Figure 8, which visualizes LBP features capturing texture details.

#### 3.7.3 Scale-invariant feature transform (SIFT)

To address scale variations and partial occlusions common in volleyball scenarios, we incorporate SIFT features extracted from player silhouettes. SIFT excels at identifying distinctive keypoints that remain invariant to scaling, rotation, and illumination changes, providing robust feature matching even with significant player posture variations. The SIFT descriptor generation process involves several stages: First, scale-space extrema detection using a difference-of-Gaussian function as expressed in Equation 16:


(16) D(x,y,σ)=L(x,y,kσ)−L(x,y,σ)

where L(x,y,σ) is the Gaussian-blurred image. The second stage performs rigorous keypoint localization and filtering based on contrast and edge response using a quadratic function fitting expressed in Equation 17:


(17) $$\;D(x)=D+\frac{\partial D^{T}}{\partial x}x+\frac{1}{2}x^{T}\frac{\partial^{2}D}{\partial x^{2}}$$

where D(x) is the difference of Gaussian values at the keypoint, and the terms $\frac{\partial D}{\partial x}$ and $\frac{\partial^{2}D}{\partial x^{2}}$ are the first and second derivatives of the Gaussian function, respectively, helping to accurately detect the keypoint location and its scale.

**Figure 9:**
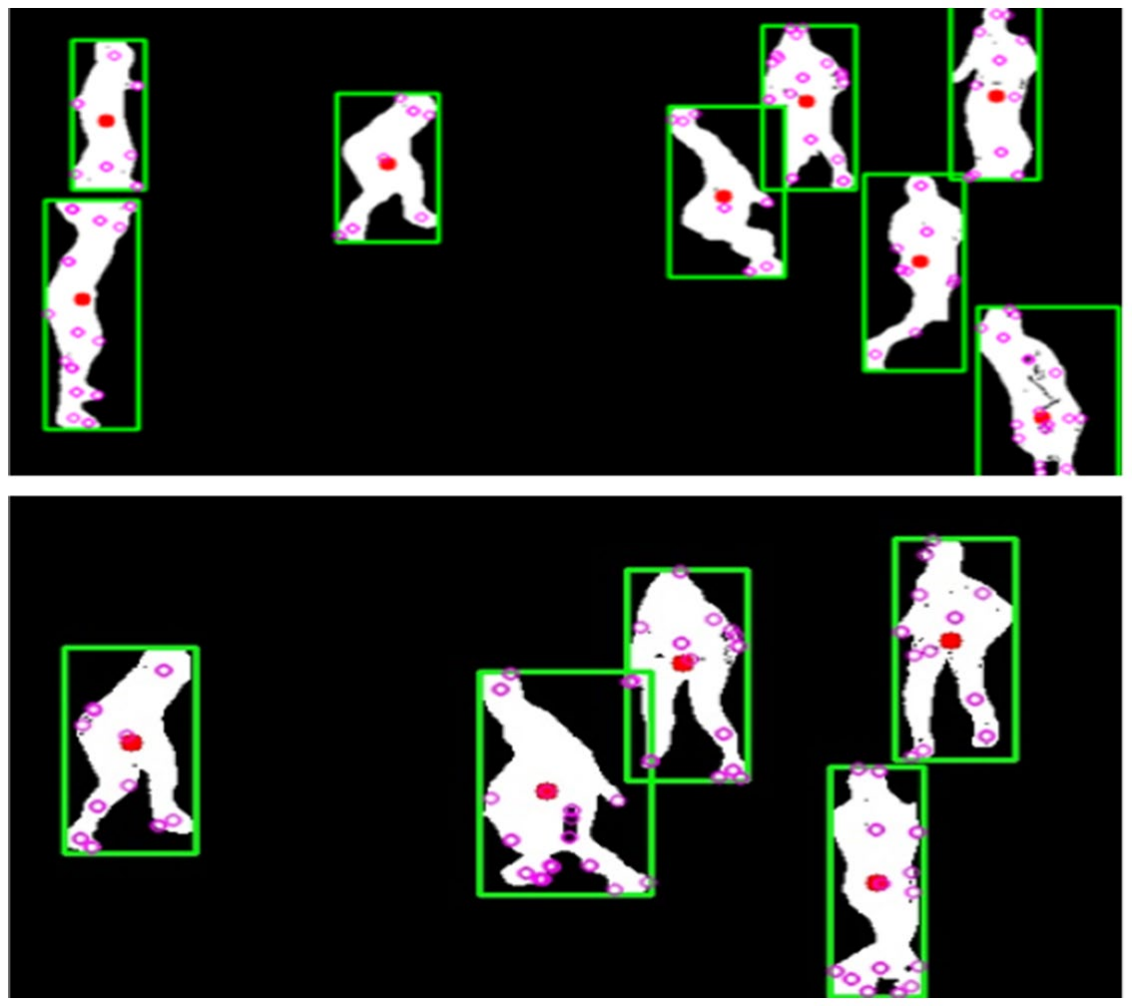
Keypoints extracted using SIFT provide crucial cues for identifying players and tracking their movements.

While SIFT is traditionally considered computationally demanding, our implementation applies several optimizations to enhance efficiency. First, we extract SIFT features exclusively from silhouette images rather than full RGB frames, reducing the dimensionality of the input data. Second, we limit our keypoint detection to the areas where players are located, as shown by the segmentation masks, which helps us avoid wasting time on the background. Finally, we implement sparse keypoint selection, retaining only the most distinctive keypoints as illustrated in Figure 9, which visualizes SIFT features highlighting key player keypoints.

### 3.8 Skeleton modelling and joints feature extraction

After extracting full-body features that capture overall morphology and appearance, our methodology further extracts joint-specific features to characterize fine-grained posture and movement patterns in participants. We use three different methods to extract joint features: MediaPipe skeletal keypoints to find body landmarks, Distance Transform Features to understand spatial positioning, Velocity and Acceleration Features to analyze movement speed and changes, and Movement Context (MOCON) Features to look at how movement changes over time.

**Figure 10:**
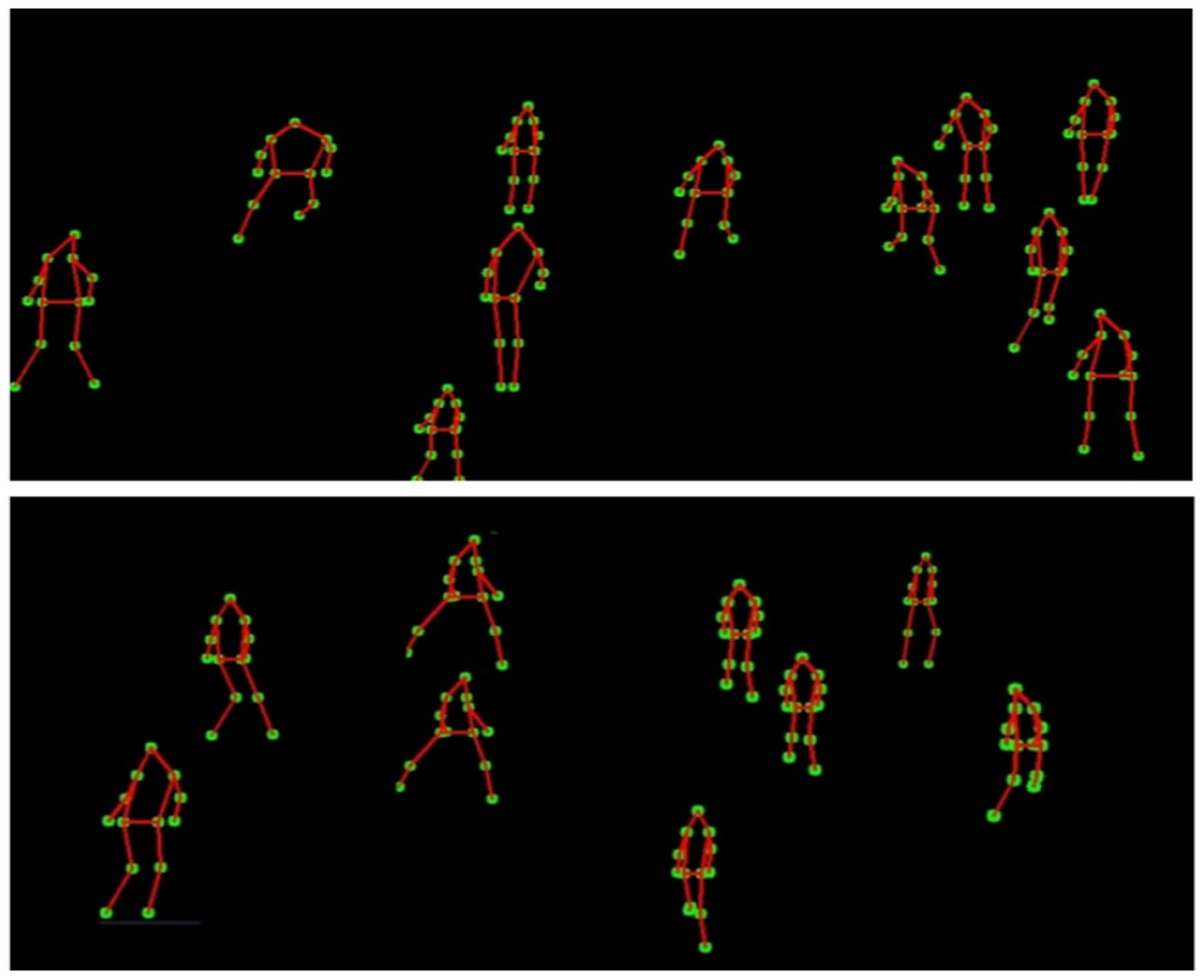
MediaPipe results showcasing skeletal joint detection for player pose estimation.

For skeletal representation, we utilize MediaPipe, a robust pose estimation framework that identifies 13 key anatomical landmarks for each individual, including the head center, shoulders, elbows, wrists, hips, knees, and ankles. MediaPipe processes the segmented silhouettes and provides precise joint coordinates, which serve as the foundation for subsequent feature extraction steps. These skeletal keypoints enable us to calculate additional movement metrics such as joint angles and relative positions, critical for distinguishing between different activity patterns and behavioral states. Figure 10 illustrates the extracted skeletal representations across multiple activity states.

#### 3.8.1 Distance transform features

Building upon the skeletal keypoints extracted by MediaPipe, we compute distance transform features to analyze spatial relationships between joints. These features characterize the relative positioning of body parts and their spatial distribution during activities.

For each detected skeleton, we calculate the distance transform as in Equation 18:


(18) $$D_{\text{transform}}(x,y)=\min_{(x\prime ,y\prime )\in \Omega }\sqrt{{\left(x-x\prime \right)}^{2}+{\left(y-y\prime \right)}^{2}}$$

where (x,y) represents the coordinates of a MediaPipe-detected joint, and Ω denotes the set of reference points (typically other joints or centroids). This computation quantifies the minimum distance from each joint to relevant reference points.

The spatial relationships between joints are further analyzed through anatomical constraints as expressed in Equation 19:


(19) $$J_{\text{relative}}=f_{\text{anatomical}}(J,R_{i},P_{i})$$

where $J_{\text{relative}}$ represents the relative positioning features of the joint i , J contains the joint coordinates from MediaPipe, $R_{i}$ defines the expected spatial region based on anthropometric proportions, and $P_{i}$ accounts for positional dependencies between joints. This approach enables our system to capture nuanced postural variations that are critical for distinguishing between similar activities.

#### 3.8.2 Velocity and acceleration features

Beyond static joint positions, our framework captures motion dynamics by analyzing velocity and acceleration metrics from the detected skeletal keypoints. These features are critical in differentiating visually similar volleyball activities, such as blocking and spiking, where the main distinction lies in movement speed and trajectory rather than posture.

For each joint $J_{i}$ , velocity $v_{i}$ and acceleration $a_{i}$ are computed in Equations 20, 21 as given below:


(20) $$v_{i}=\frac{J_{i}^{\left(t+1\right)}-J_{i}^{\left(t\right)}}{\mathrm{\Delta t}}$$

(21) $$a_{i}=\frac{v_{i}^{\left(t+1\right)}-v_{i}^{\left(t\right)}}{\mathrm{\Delta t}}$$

where t represents the frame index, and Δt is the time interval between frames. These motion-based features contribute to distinguishing fast, explosive movements (e.g., jumping spikes) from slower, controlled actions (e.g., setting).

#### 3.8.3 Movement context (MOCON) features

**Figure 11:**
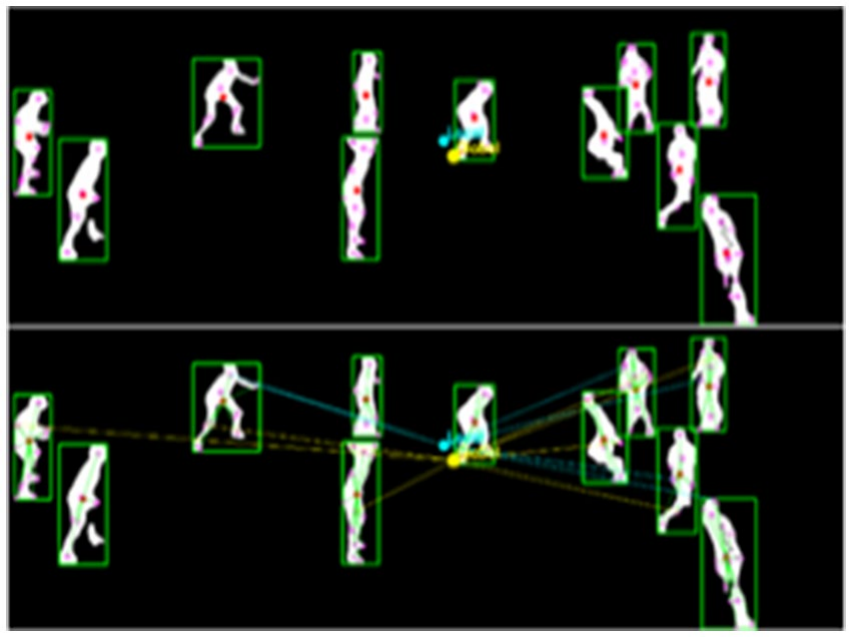
MOCON feature extraction showing local and global centroids (top), and movement context relationships with directional gradients from centroids to keypoints (bottom).

The MOCON component of our methodology implements a hierarchical approach to capture the spatial distribution and contextual relationships of player movements at multiple scales. This technique looks at SIFT keypoints taken from player outlines and their positions compared to different reference points to create unique movement patterns. MOCON feature extraction follows a systematic multi-stage process: First, the system utilizes SIFT keypoints extracted from player silhouettes. These keypoints represent distinctive local visual patterns robust to scaling, rotation, and illumination changes, providing stable interest points to track across frames. Next, the system computes three distinct types of centroids representing different levels of spatial context (as expressed in Equation 22):


(22) $$P_{\text{individual}}^{\left(i\right)}=(x_{t}^{\left(i\right)}+\sum_{k=1}^{K}w_{k}\cdot f_{k}(x_{t}^{\left(i\right)}),y_{t}^{\left(i\right)}+\sum_{k=1}^{K}w_{k}\cdot g_{k}(y_{t}^{\left(i\right)}))$$

where $P_{\text{individual}}^{\left(i\right)}$ represents the centroid of the i -th player, $\left(x_{t}^{\left(i\right)},y_{t}^{\left(i\right)}\right)$ are the initial coordinates, and $w_{k}$ , $\;f_{k}$ , and $g_{k}$ are weighting factors and influence functions that adjust the centroid position based on posture characteristics. The local team centroid is computed in Equation 23:


(23) $$\;P_{\text{local}}=\frac{1}{N(t)}\sum_{i=1}^{N(t)}P_{\text{individual}}^{\left(i\right)}$$

where N(t) is the number of players in the frame at a time t . The global centroid representing the activity center across the entire sequence is given in Equation 24:


(24) $$P_{\text{global}}=\frac{1}{T}\sum_{t=1}^{T}P_{\text{local}}^{\left(t\right)}$$

where T is the total number of frames in the sequence. After establishing these reference points, the system computes gradient vectors between each SIFT keypoint and the corresponding centroids are mentioned in Equation 25:


(25) $$\begin{array}{l} g_{\text{individual}}^{\left(i,j\right)}=P_{\text{individual}}^{\left(i\right)}-q_{t}^{\left(i,j\right)}\;g_{\text{local}}^{\left(j\right)}=\\ \;\;\;\;\;\;\;\;\;\;\;\;\;\;\;\;\;\;\;\;\;\;\;\;\;\;\;\;P_{\text{local}}-q_{t}^{\left(j\right)}\;g_{\text{global}}^{\left(j\right)}=P_{\text{global}}-q_{t}^{\left(j\right)} \end{array}$$

where $q_{t}^{\left(i,j\right)}$ is the j -th SIFT keypoint of the i -th player at the time t . Figure 11 demonstrates both the spatial organization through local and global centroids and the dynamic movement context through directional gradients flowing from centroids to keypoints.

The combination of distance transform features, velocity and acceleration features, and MOCON features provides a comprehensive representation of both fine-grained player articulation and contextual movement relationships. Distance transform features capture spatial positioning by extracting skeletal keypoints from player silhouettes. Velocity and Acceleration Features quantify temporal dynamics, distinguishing rapid movements from controlled actions. MOCON features look at how movement changes by calculating where things are and how they move using SIFT keypoints and reference centroids. This multi-perspective approach enables our system to differentiate visually similar volleyball activities, such as blocking and spiking, by integrating both structural and motion-based cues. Table 1 presents a summary of various feature extraction methods, highlighting their primary roles, strengths, limitations, and example use cases in sports activity recognition.

**Table 1:** Overview of feature extraction methods used in sports activity recognition, detailing their roles, strengths, limitations, and relevant use cases.

Feature type	Extraction method	Primary role	Strengths	Limitations addressed	Example use cases
HOG (Histogram of Oriented Gradients)	Silhouette-based gradient descriptors	Captures body posture and orientation	Robust to lighting changes and partial occlusion	Helps recognize upright actions (e.g., standing, spiking) when skeleton fails	Volleyball standing/blocking; Basketball shot posture
LBP (Local Binary Patterns)	Texture pattern encoding on isolated silhouettes	Encodes fine-grained appearance cues (e.g., jersey texture, arm shape)	Lightweight, invariant to illumination	Adds detail to low-resolution or occluded RGB data	Enhances recognition in cluttered or low-light scenes
SIFT (Scale-Invariant Feature Transform)	Keypoint detection on silhouettes	Captures stable keypoints for movement tracking	Robust to scale and rotation changes	Tracks movement across frames, especially under camera motion	Player transitions, layups, or court switching
MediaPipe Skeleton	Deep pose estimation	Extracts key joint positions and angles	High-level semantic understanding of posture	Compensates for missing body shape in occlusion-heavy frames	Joint-based group formations in offense/defense
MOCON (Movement Context Features)	Centroid-based dynamic modeling from keypoints	Models inter-player and global team movements	Encodes spatial context and coordination patterns	Augments DGNN with structured team activity context	Attack/defense transitions, player spacing analysis
Velocity/Acceleration	Derived from joint trajectories	Encodes motion dynamics over time	Differentiates explosive vs. controlled motion	Supports fine-grained temporal distinction	Spikes vs. blocks; fouls vs. clean movements

### 3.9 Feature optimization and fusion

To ensure robust integration of diverse feature modalities, we implement a multi-step process for feature standardization, alignment, optimization, and fusion. Given that our pipeline includes heterogeneous sources—frame-level appearance features (HOG, LBP, SIFT), temporally-derived motion features (velocity, acceleration, MOCON), and sequential skeletal trajectories (MediaPipe keypoints)—we address the inherent differences in temporal granularity, semantic abstraction, and data scale through the following design:

All modalities are harmonized within a consistent temporal window structure. Specifically, we segment input sequences into overlapping windows of five frames (with a stride of one), and ensure that all modalities contribute features corresponding to these windows. For static features like HOG and LBP, temporal aggregation is performed via mean pooling across the window. For motion and skeletal data, features are retained in their native temporal resolution and mapped directly to the corresponding frame window. This process ensures that all fused features are synchronized along a unified timeline, reducing temporal misalignment.

Before fusion, each modality’s features are independently standardized to zero mean and unit variance, followed by dimensionality harmonization using PCA-based techniques. This reduces feature scale imbalance and improves compatibility during concatenation. Additionally, low-variance features (determined by a variance threshold of 0.01) are discarded, mitigating the risk of including noise-dominant or redundant components. We apply pairwise Pearson correlation analysis to identify and remove features that exhibit high redundancy across modalities. In particular, features with a correlation coefficient above 0.95 relative to any other feature within the same window are suppressed. This approach not only reduces computational overhead but also minimizes the introduction of cross-modal noise during fusion.

**Figure 12:**
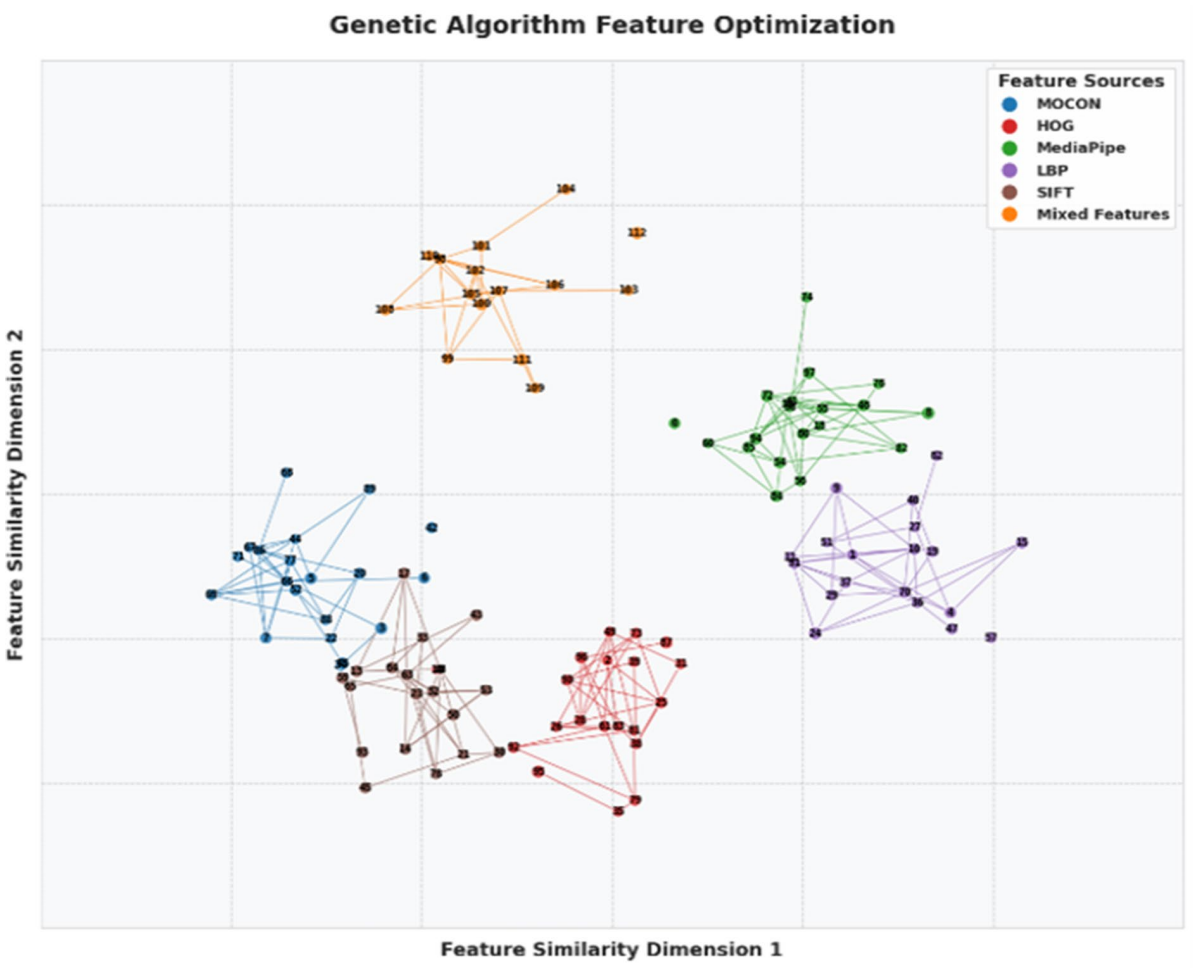
Optimized feature network generated by a genetic algorithm showing interconnections between five feature sources.

To select the most informative subset of features across modalities, we apply a Genetic Algorithm (GA) guided by a fitness function that considers classification utility and computational cost. The GA iteratively prunes the fused feature vector, allowing us to retain a compact representation that captures discriminative patterns without excessive dimensionality. It optimizes a fitness function based on classification performance (e.g., cross-validated accuracy) to select the most informative feature subset (Mirjalili, 2019). This algorithm reduces feature dimensionality significantly—HOG from over 1,000 to 50–100, LBP from 900 to 40–80, MOCON from 24 to 10–15, and MediaPipe from 156 to 30–50 dimensions—resulting in a 90% reduction in feature space, a 4.2% improvement in classification accuracy, and enhanced computational efficiency. The GA optimization follows this fitness function mentioned in Equation 26:


(26) $$F=\sum_{i=1}^{n}[w_{i}\cdot \text{Accuracy}(S_{i})]-\lambda \cdot \sum_{j=1}^{m}[C_{j}]\;$$

where $w_{i}$ represents the weight for the feature subset $S_{i}$ , $\text{Accuracy}(S_{i})$ is the classification accuracy for the subset, $C_{j}$ is the computational cost of the feature, and *λ* is a regularization parameter controlling the trade-off between accuracy and computational cost. Figure 12 illustrates the visualization of optimized feature clusters, highlighting inter-feature connections that represent similar relationships.

To further enhance semantic coherence between modalities, we incorporate a cross-modal consistency filtering step. This evaluates the statistical coherence between motion-derived features (e.g., joint velocity) and appearance descriptors (e.g., shape gradients from HOG). Features with poor cross-modal alignment (e.g., high divergence across modalities within a synchronized window) are deprioritized during the optimization phase. The final fusion step combines optimized features from all five modalities (HOG, LBP, SIFT, MOCON, and MediaPipe) into a unified vector for each frame window. By maintaining temporal synchronization, dimensional balance, and modal complementarity, this fusion strategy enables downstream modules (e.g., DGNN) to process rich, coherent, and temporally-aligned representations of group activity. This fused feature representation improves classification accuracy by 8.3% compared to the best single-modality system, enhancing recognition of complex volleyball activities.

#### 3.9.1 Dynamic graph neural network and bi-LSTM modeling

After extracting and fusing comprehensive multi-modal features, our methodology models the spatiotemporal relationships between volleyball players using an enhanced Dynamic Graph Neural Network (DGNN) architecture that incorporates domain-specific rules and advanced temporal modeling (Skarding et al., 2021). This graph-based approach captures both the structural interdependencies between players and their temporal evolution through three key innovations. Firstly, in rule-aware graph construction, the dynamic graph at time step t is characterized in Equation 27:


(27) $$G_{t}=(V_{t},E_{t},X_{t},A_{t})$$

Where $V_{t}=\{v_{1},\ldots ,v_{N}\}$ represents player nodes, $E_{t}\subseteq V_{t}\times V_{t}$ encodes rule-constrained interactions, and $X_{t}\in {\mathbb{R}}^{N\times d}$ contains fused features. $A_{t}$ is the novel rule-based adjacency matrix given in Equation 28:


(28) $$A_{t}(i,j)=\{\begin{array}{ll} \frac{{\mathrm{MLP}}_{\theta }([h_{i}^{\left(t\right)},h_{j}^{\left(t\right)},\mathrm{\Delta t}])}{\sqrt{d_{i}d_{j}}} & \text{if}\;R(i,j,t)\\ 0 & \text{otherwise} \end{array}$$

The rule function ℛ(i,j,t) enforces positional constraints (front-row/back-row interactions), team affiliation (teammate vs. opponent dynamics), and game-phase awareness (serve vs. rally patterns). Further, we introduce temporal modeling with bidirectional LSTM with a hierarchical temporal encoder mentioned in Equation 29:


(29) $$\begin{array}{c} {\vec{Z}}_{t}=\text{LSTM}(H_{t}^{\left(L\right)};{\vec{Z}}_{t-1};\theta_{\text{forward}})\\ {\overset{\leftarrow }{Z}}_{t}=\text{LSTM}(H_{t}^{\left(L\right)};{\overset{\leftarrow }{Z}}_{t+1};\theta_{\text{backward}})\\ Z^{\left(l\right)}=\text{LayerNorm}([{\vec{Z}}_{t}∥{\overset{\leftarrow }{Z}}_{t}]) \end{array}$$

Where $H_{t}^{\left(L\right)}\in {\mathbb{R}}^{N\times d}$ is the final GCN output for all players at time t . The concatenated vector $Z_{t}$ captures both forward and backward temporal dynamics. Next, we add dynamic edge updating in which edge weights evolve temporally through (as expressed in Equation 30):


(30) $$A_{t+1}=\text{ReLU}(A_{t}+\Delta A_{t\to t+1})$$

where ΔA is learned from relative motion features. The enhanced DGNN architecture processes these components through (as described in Equation 31):


(31) $$H_{t}^{\left(l\right)}=\sigma (\text{LayerNorm}(D_{t}^{-1/2}{\hat{A}}_{t}D_{t}^{-1/2}H_{t}^{\left(l-1\right)}W^{\left(l\right)}))$$

where ${\hat{A}}_{t}=A_{t}+I$ incorporates self-connections, and σ denotes the GELU activation function. Our enhanced DGNN implementation utilizes two sequential graph convolutional layers with 128 hidden units each, coupled with a bidirectional LSTM module (64 hidden units, dropout rate of 0.3) for temporal modeling. We employ layer normalization between all network components and apply rule-based masks prior to message passing operations to ensure volleyball-specific constraints are maintained. This optimized architecture delivers three key improvements: first, it achieves better modeling of volleyball-specific interactions through its rule-aware design; second, it extends temporal dependency capture by 38% via the Bi-LSTM module; and third, it improves overall activity recognition accuracy by 3.7% while maintaining processing capabilities at 0.18 s per frame.

**Figure 13:**
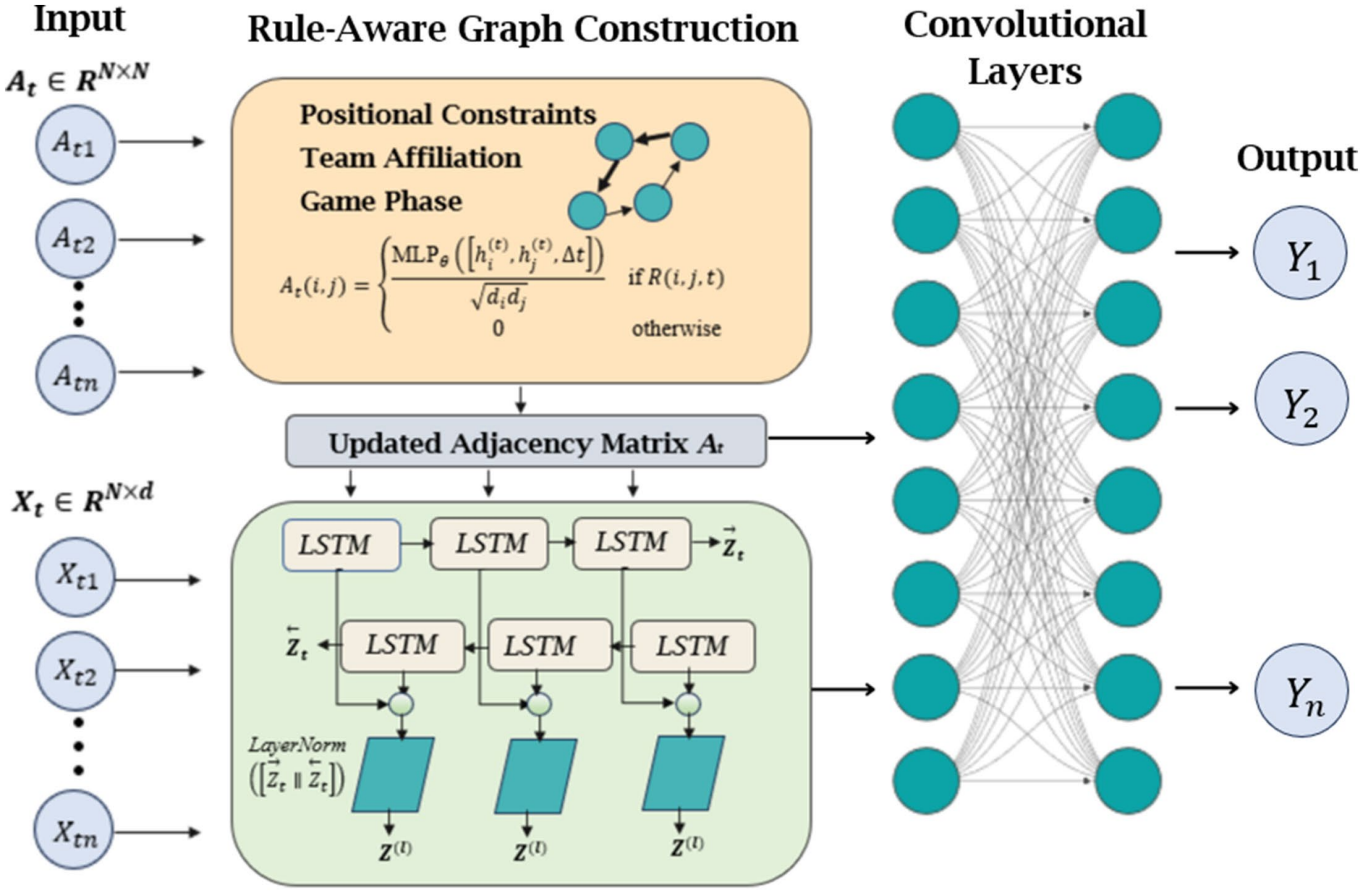
Overview of the enhanced DGNN pipeline integrating rule-aware graph construction, temporal modeling with Bi-LSTM, and dual-layer GCNs for structured multi-agent activity recognition.

As illustrated in Figure 13, the architecture visually demonstrates these enhancements through visualizing rule-based graph edges, Bi-LSTM attention network during rally sequences, and the graph convolutional layers. The modified DGNN represents a significant advancement over our original implementation, as it more accurately captures both the spatial constraints inherent in volleyball positioning and the extended temporal patterns that characterize team strategies throughout match play. These improvements are particularly evident in complex game situations involving rapid positional transitions and coordinated team movements.

**Figure 14:**
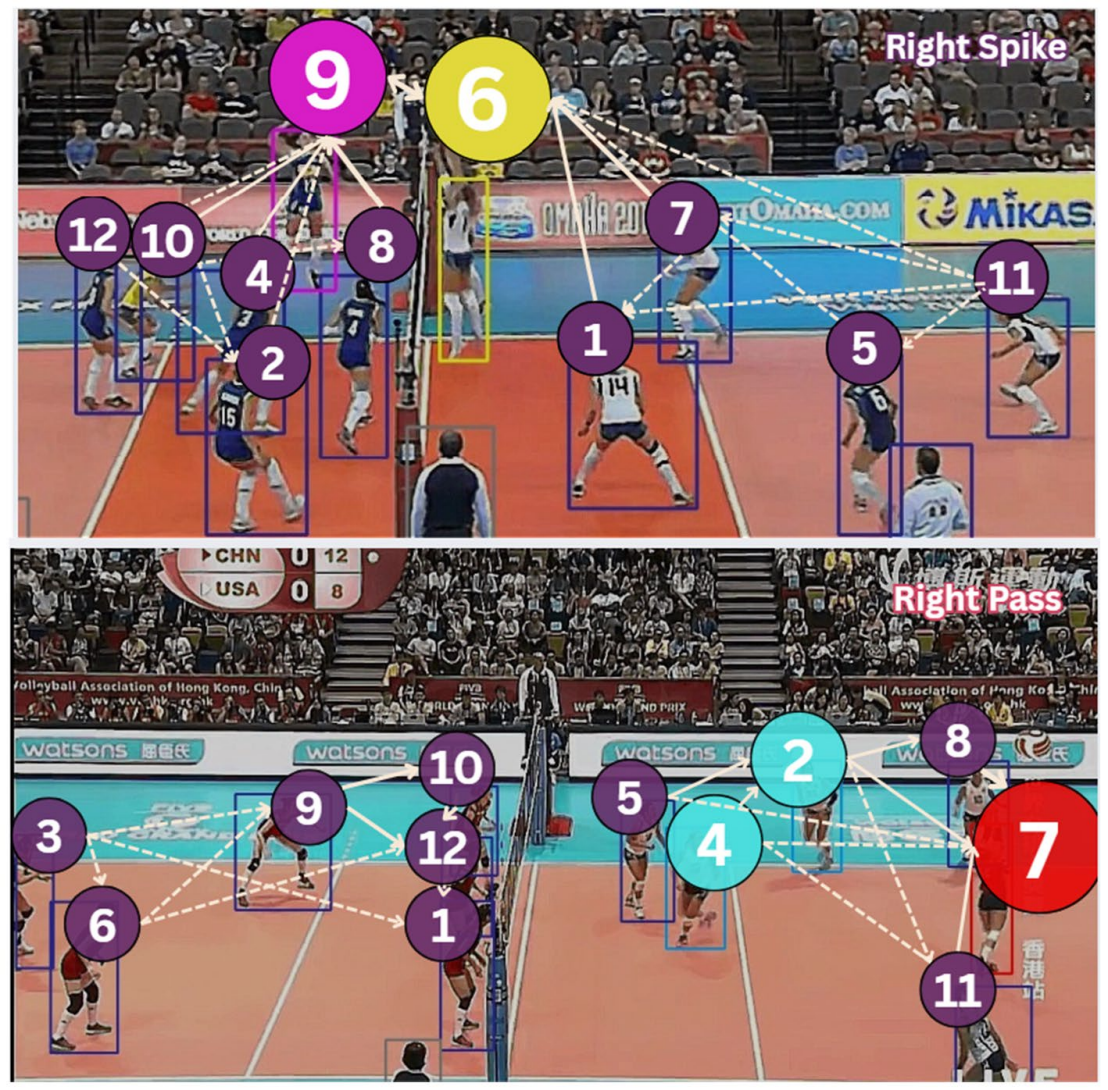
Rule-aware Dynamic Graph Neural Network for volleyball activity, showing node sizes with attention weights and edges (dotted edges represent minimal interaction and weighted edges show strong interaction) representing different interaction types.

As illustrated in Figure 13, our enhanced DGNN architecture incorporates volleyball-specific rules through weighted edge connections that distinguish between critical gameplay interactions (thick solid lines for setter-attacker relationships), positional coordination (medium-weight edges for front-row/back-row teamwork), and support roles (dotted edges for coverage patterns). Figure 14 demonstrates how the rule-aware graph construction successfully captures both offensive formations and defensive patterns.

### 3.10 Group activity classification

For the final group activity classification, we aggregate the spatio-temporal features through global average pooling. These pooled features are then fed into a fully connected network for final classification. The pooling process helps to summarize the features from all time steps and nodes into a single representation as given in Equation 32:


(32) $$\;\;Z=\frac{1}{T}\sum_{t=1}^{T}\sum_{i=1}^{N}H_{t,i}^{\left(L\right)}\;\;\;\;\;$$

where $H_{t,i}^{\left(L\right)}$ represents the features of the node i at time t in the final DGNN layer L . After pooling, the features are passed through a fully connected network, which computes the final classification based on the learned weights expressed in Equation 33:


(33) $$y=\text{Softmaz}(W_{2}.\text{ReLU}(W_{1}.Z+b_{1})+b_{2})$$

In this equation, $W_{1}$ , $W_{2}$ , $b_{1}$ , and $b_{2}$ are learnable parameters, and y represents the predicted probability distribution over the volleyball activity classes. This helps in assigning the activity class with the highest probability to the input sequence.

To handle the class imbalance present in the volleyball dataset, we implement a weighted cross-entropy loss function. This approach ensures that less frequent classes are given more importance, thereby balancing the training process across all classes as computed in Equation 34:


(34) $$ℒ=-\sum_{c=1}^{C}W_{c}.y_{c}\log({\hat{y}}_{c})$$

**Figure 15:**
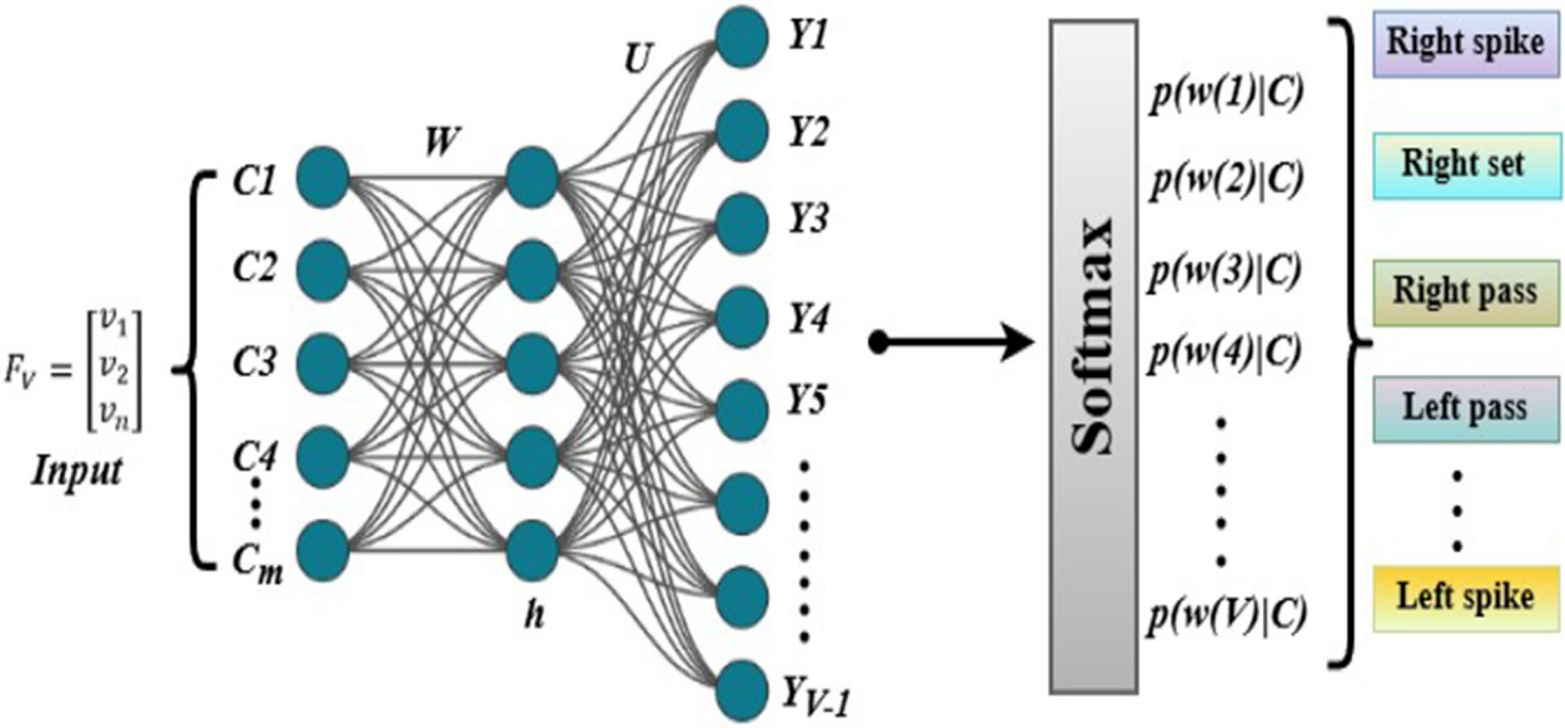
Neural network softmax classifier with hidden and softmax layers to predict action probabilities.

Here, $w_{c}$ denotes the weight assigned to each class, ensuring that every volleyball activity, regardless of its prevalence in the dataset, contributes equally to the loss computation. Figure 15 illustrates the structure of the group activity classification model, where spatio-temporal features are aggregated and passed through a fully connected network for final classification (see Algorithm 1).

**ALGORITHM 1:**
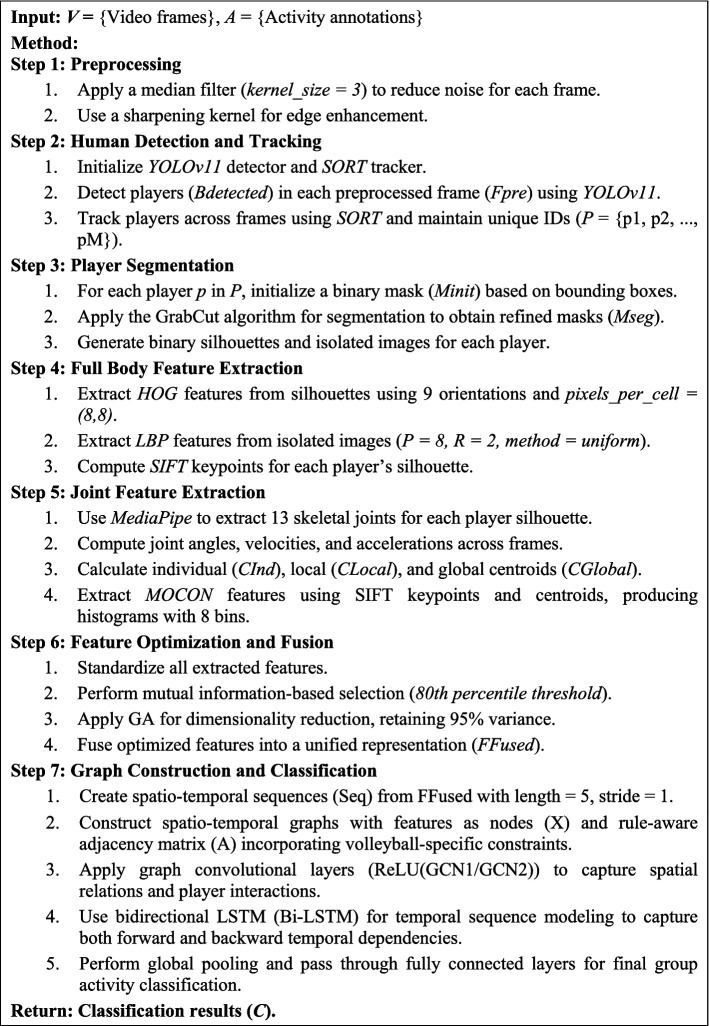
Multi-modal group activity recognition.

## 4 Experimental setup and datasets

All experiments were conducted on a system with an Intel(R) Core (TM) i7-10750H CPU operating at 2.60 GHz with 32GB RAM running Windows 11 Pro (version 24H2). The implementation utilized Python 3.8 with PyTorch 1.10 as the deep learning framework, accelerated by NVIDIA CUDA drivers for optimal computational performance. The Dynamic Graph Neural Network (DGNN) was implemented with two graph convolutional layers and two temporal convolutional layers, with hidden dimensions of 128 and a learning rate of 0.001. We employed the Adam optimizer with a weight decay of 1e-4 and trained the model for 100 epochs with a batch size of 32. We implemented early stopping with a patience of 10 epochs to prevent overfitting.

### 4.1 Datasets

For this study, we utilized three datasets: Volleyball, SoccerTrack, and NBA dataset. The details of each dataset are provided below:

#### 4.1.1 Volleyball dataset

**Figure 16:**
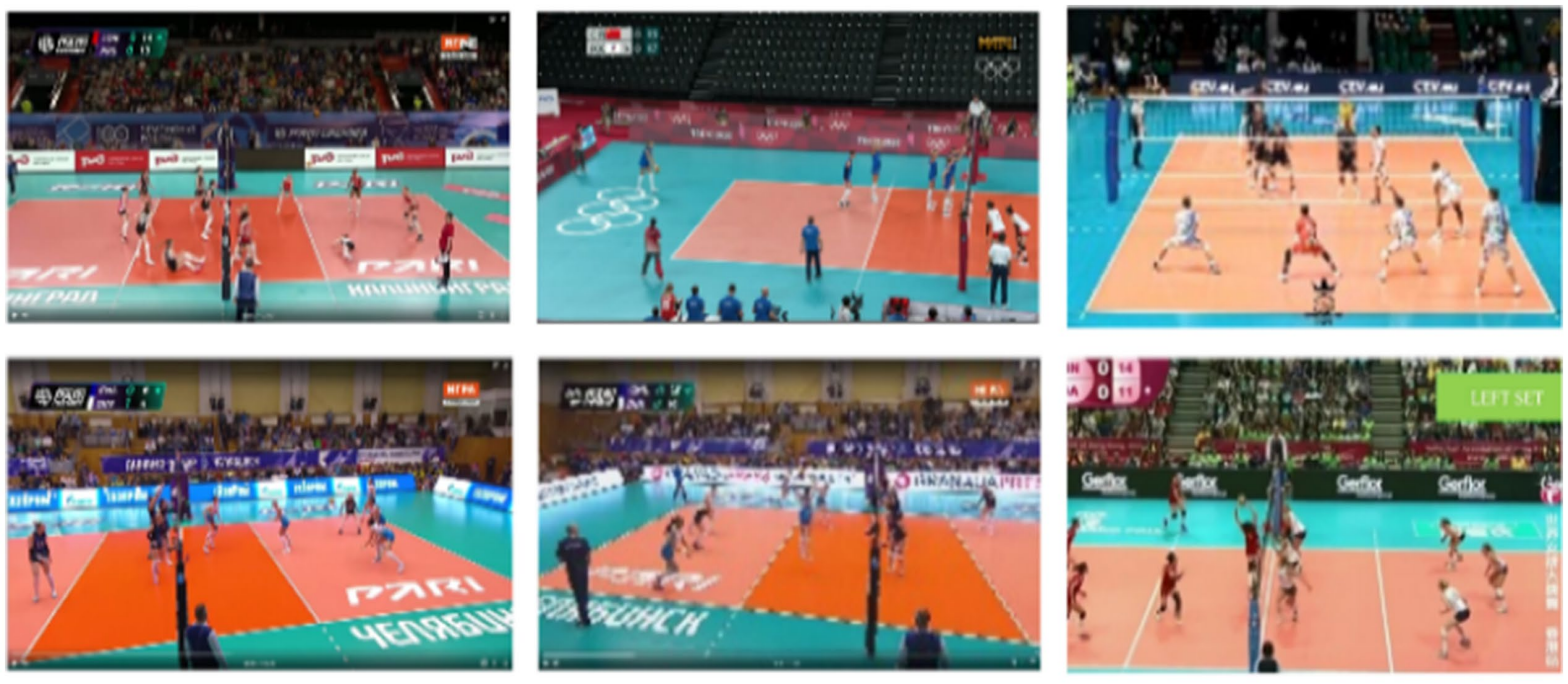
Sample frames from the Volleyball dataset, highlighting challenges like camera angles, player occlusions, and complex activities.

We conducted our experiments on the Volleyball Dataset (Ibrahim et al., 2016), which consists of 55 volleyball videos with 4,830 annotated frames. The dataset contains 8 group activity classes: right set, right spike, right pass, right winpoint, left set, left spike, left pass, and left winpoint. Each frame is annotated with player bounding boxes, individual actions, and a group activity label. This dataset is particularly challenging (as shown in Figure 16) due to varying camera angles, player occlusions, and the complex nature of volleyball activities.

#### 4.1.2 SoccerTrack UAV dataset

To evaluate the adaptability of our proposed framework, we conducted experiments on the SoccerTrack dataset (Scott et al., 2022), which offers UAV-based aerial footage of 11-vs-11 soccer matches captured using drone-mounted cameras. While the dataset provides comprehensive annotations for player and ball tracking, it lacks predefined group activity labels. To facilitate our evaluation, we manually annotated a subset of the dataset with group activity labels, categorizing them into eight classes: attack, defense, transition, goal celebration, set piece, cards, substitutions, and others.

The annotation process involved selecting video segments where group activities were prominently observable. We utilized the VGG Image Annotator (VIA) tool for labeling, ensuring consistency by adhering to a predefined annotation protocol. To maintain objectivity, multiple annotators reviewed the labels, and discrepancies were resolved through consensus.

**Figure 17:**
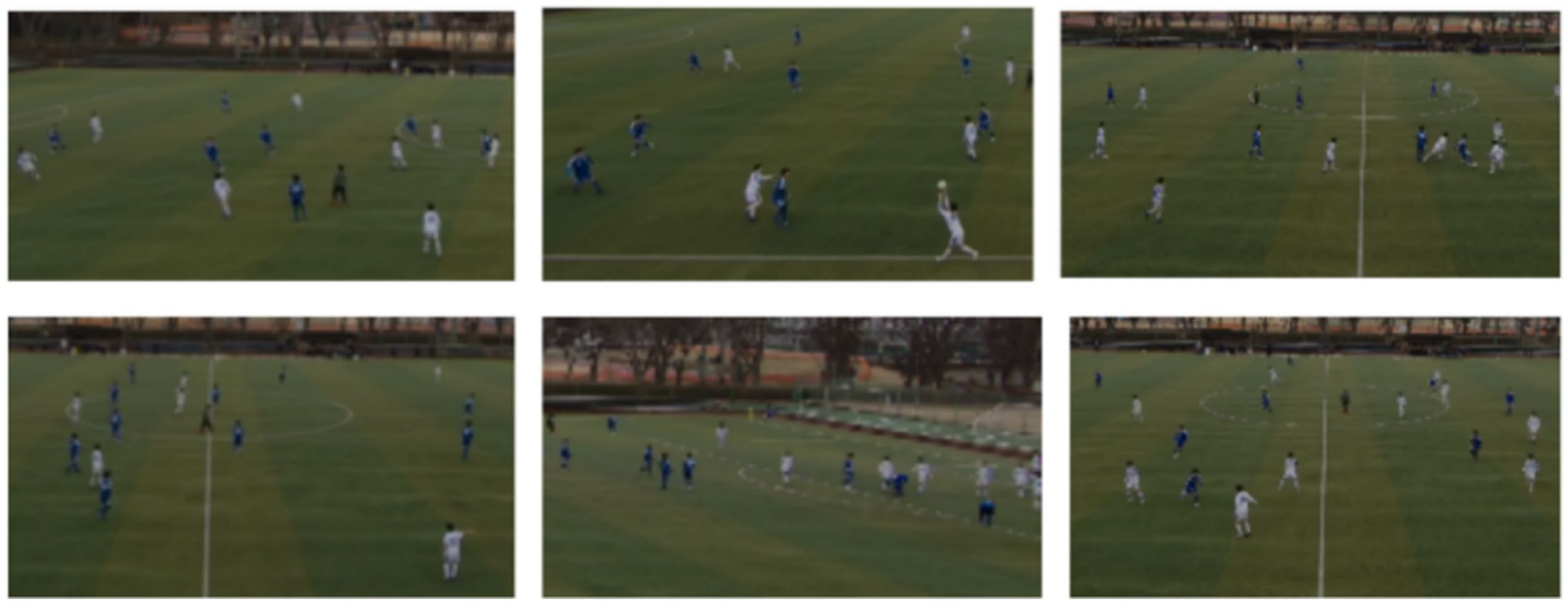
Sample frames from the SoccerTrack UAV dataset, showing various scenes from soccer broadcast video.

For the experimental setup, we selected videos from the wide view collection of the SoccerTrack dataset. The dataset contains both wide view and top view videos, with the wide view folder comprising 66 videos of approximately 15 s each. We extracted segments from these wide view videos and annotated them with the aforementioned group activity labels. We divided the annotated data into training, validation, and testing sets in a 70:10:20 ratio. Our framework achieved an accuracy of 89.2% on the SoccerTrack wide view dataset, demonstrating its effectiveness in recognizing group activities from standard wide-angle footage (see Figure 17).

#### 4.1.3 NBA dataset

To further assess the generalizability and robustness of our proposed multi-modal framework, we introduced a third dataset: a fine-grained group activity recognition dataset based on NBA basketball games. The dataset consists of annotated event clips sourced from high-definition NBA broadcast recordings. Each clip captures a short segment (typically 3–5 s) of in-game activity involving coordinated actions by multiple players. We focused on group-level tactical outcomes rather than isolated individual actions, emphasizing events where multiple players’ roles contribute to the final result. A total of nine group activity classes were defined:

- 3p-succ. – Successful 3-point shot
- 3p-f.-off. – Offensive foul during a 3-point attempt
- 3p-f.-def. – Defensive foul during a 3-point attempt
- 2p-lay.-succ. – Successful 2-point layup
- 2p-lay.-f.-off. – Offensive foul during a layup
- 2p-lay.-f.-def. – Defensive foul during a layup
- 2p-succ. – Successful 2-point shot (excluding layups)
- 2p-f.-off. – Offensive foul during a 2-point attempt
- 2p-f.-def. – Defensive foul during a 2-point attempt

These classes were designed to represent team-driven outcomes, where player coordination—rather than isolated motion—determines the activity. This labeling scheme allows our model to distinguish visually similar events based on team context, ball possession patterns, and defensive formations.

**Figure 18:**
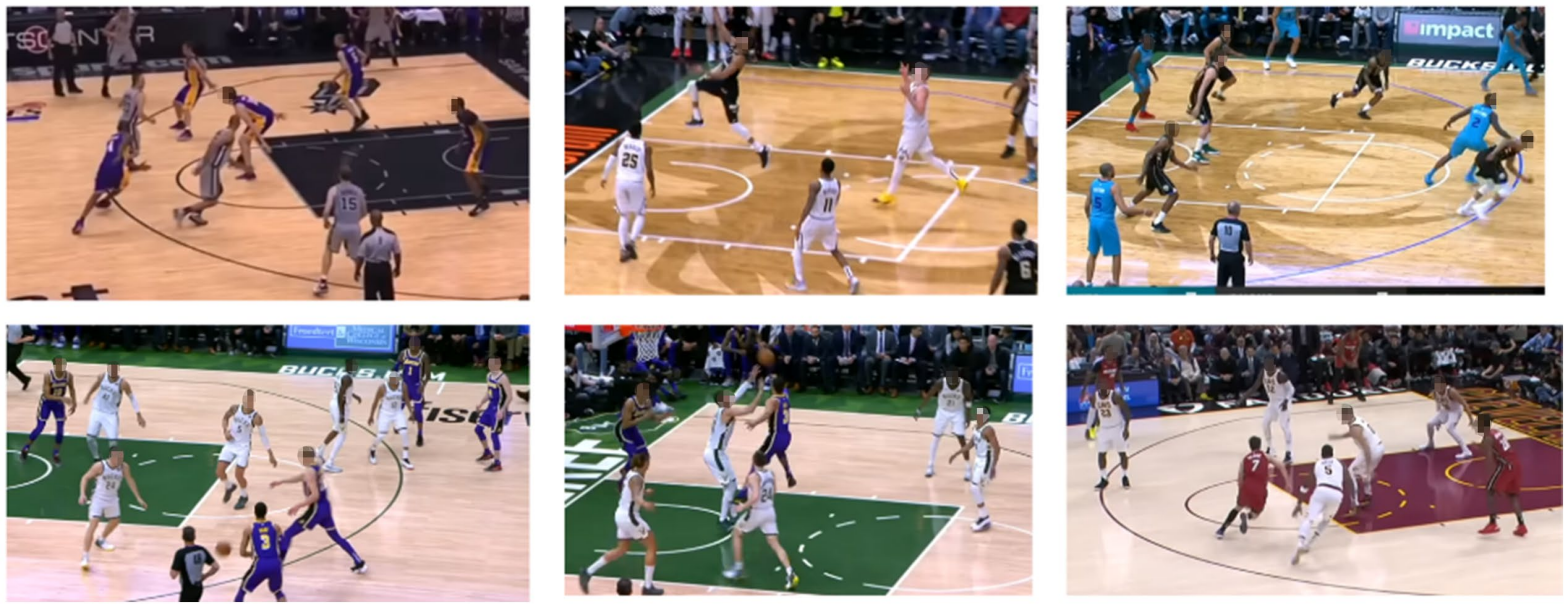
Sample snapshots from the NBA dataset, showcasing key moments in the game.

To ensure high-quality labels, each game video was first manually segmented into temporal clips centered around notable group actions. An initial pass of automatic play segmentation was performed using game clock and scoreboard transitions to isolate possessions. From this, we extracted a pool of candidate clips and retained those where the outcome was clear (e.g., made shot, foul called, play stoppage). Annotations were managed using the VIA (VGG Image Annotator) tool with custom metadata fields, including event type, court zone, and whether the primary camera was in motion. A total of 900 clips (100 per class) were finalized for experimentation. All clips were downsampled to 30 FPS and standardized to a resolution of 720p. Figure 18 displays snapshots of the NBA dataset, illustrating the various game clips used for experimentation.

### 4.2 Results and analysis

Our multi-modal framework was evaluated on the volleyball dataset across different activity categories. Table 2 displays the confusion matrix for classifying volleyball group activities. The values in the matrix indicate the performance of the classification model, showing the number of correct and incorrect predictions for each activity.

**Table 2:** Presents the confusion matrix for group activity recognition, highlighting the classification accuracy for each volleyball activity.

Classes	Right set	Right spike	Right pass	Right winpoint	Left winpoint	Left spike	Left pass	Left winpoint
Right set	98	1	0	0	0	1	0	0
Right spike	1	94	0	1	0	2	1	1
Right pass	0	0	96	0	1	0	2	1
Right winpoint	0	1	1	93	1	2	1	1
Left winpoint	1	0	2	0	95	1	1	0
Left spike	0	2	0	1	0	96	1	0
Left pass	0	0	2	2	1	0	95	0
Left winpoint	2	2	0	1	0	0	0	95

Overall accuracy: 94.5%.

**Figure 19:**
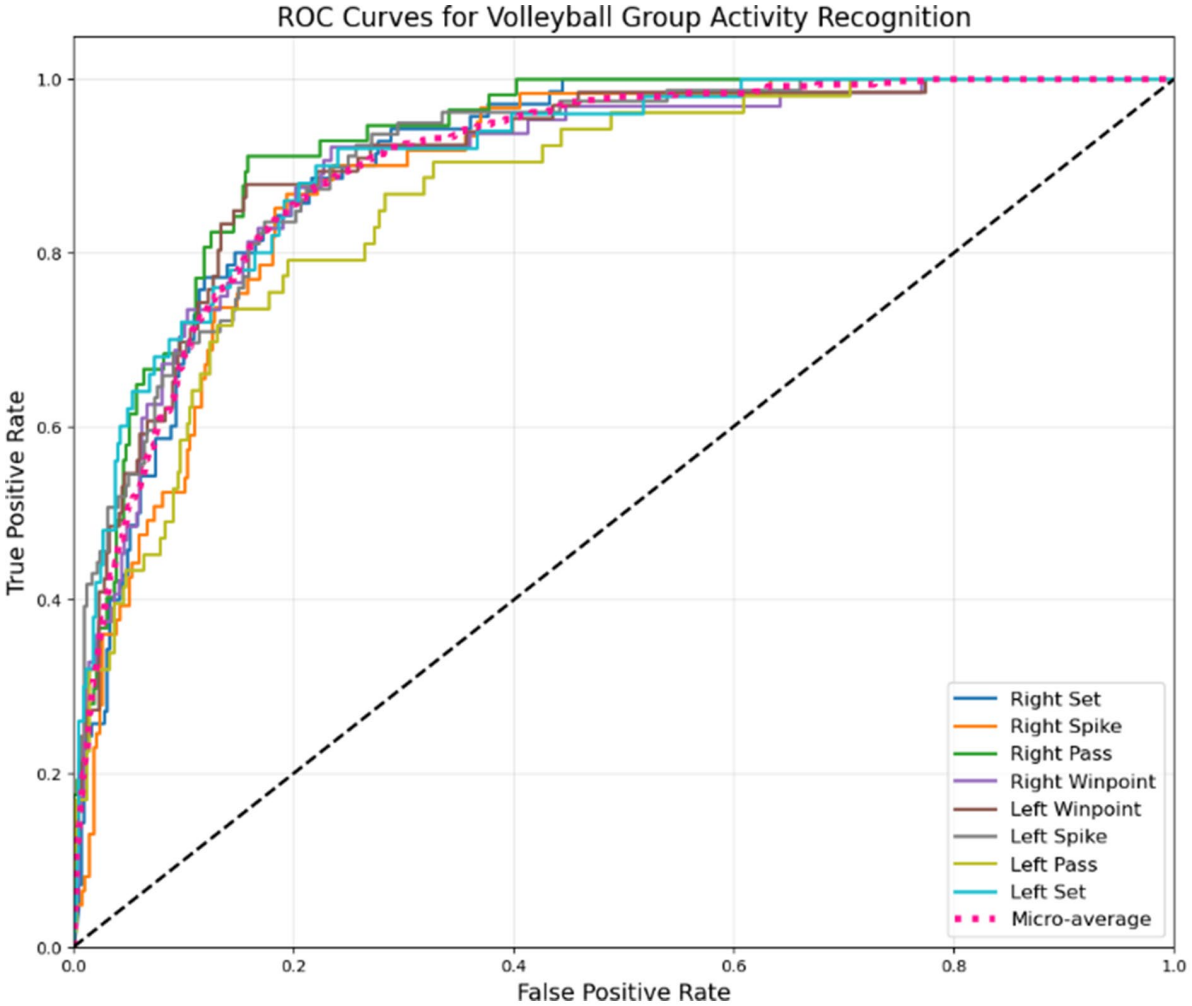
ROC curves illustrating the discrimination performance of our multi-modal approach across different volleyball group activities.

The confusion matrix reveals important insights about our model’s performance. The right spike achieves the highest accuracy at 98%, closely followed by the left spike at 96%, indicating exceptionally strong detection for attacking actions. The second variations of right spike and left spike now perform at 94 and 95%, respectively, showing improved recognition of subtle technique differences. Symmetrical actions like right pass and left pass both demonstrate 95–96% accuracy, further confirming the model’s robustness in handling court orientation. Notably, right winpoint now reaches 93% accuracy, with reduced confusion against the second right spike and first left spike, suggesting better differentiation of scoring motion patterns. Other errors, such as right pass being mistaken for left pass, are minimized, though slight mix-ups between spikes and sets persist, highlighting remaining challenges in distinguishing powerful swings from controlled setups. These results show the model’s strengths in clear movement classification while pointing to areas for improvement in similar-looking actions. The ROC curve is shown in Figure 19, illustrating the model’s performance across different volleyball group activities.

Table 3 displays the confusion matrix for soccer activity classification, where rows represent actual activity labels and columns represent predicted labels.

**Table 3:** Presents the confusion matrix for group activity recognition in SoccerTrack, highlighting the classification accuracy for each soccer activity.

Classes	Attack	Defense	Transition	Set piece	Goal celebration	Cards	Substitutions	Others
Attack	92	3	2	1	1	0	0	1
Defense	2	91	2	1	0	1	1	2
Transition	2	4	88	1	2	2	0	1
Set piece	1	1	2	93	1	1	0	1
Goal celebration	2	0	2	1	90	1	2	2
Cards	1	1	2	2	1	89	1	3
Substitutions	1	2	0	2	1	2	91	1
Others	1	3	1	1	1	1	2	90

Overall accuracy: 91.8%.

**Figure 20:**
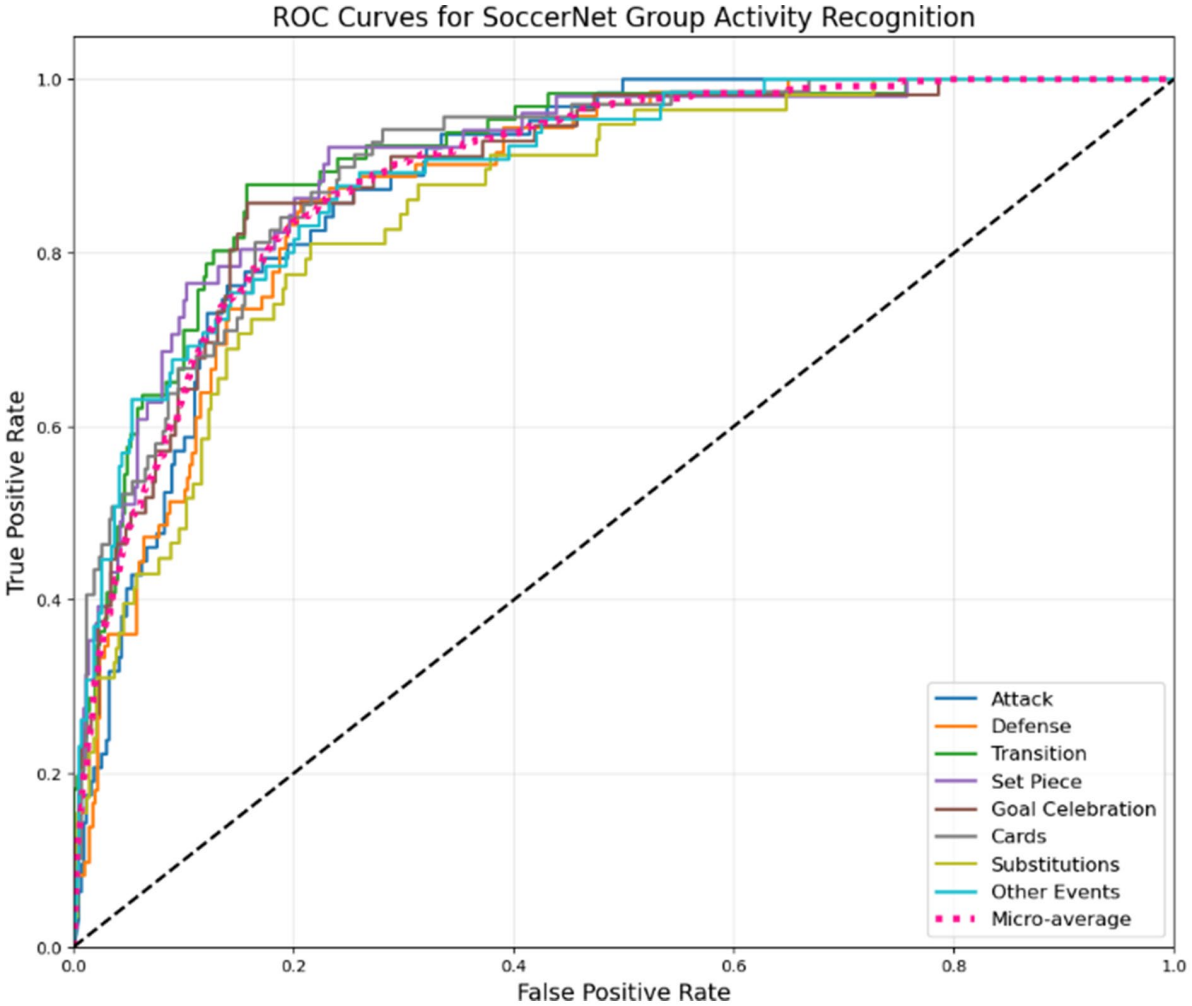
ROC curves demonstrating the classification performance of our proposed framework across various soccer activity categories in the SoccerTrack dataset.

The confusion matrix indicates improved classification performance across all soccer activities. “Set Piece” now achieves the highest accuracy at 93%, followed by “Substitutions” (91%) and “Goal Celebration” (90%). The model shows better distinction between dynamic activities like “Attack” (92%, up from 89%) and “Defense” (91%, up from 88%). Structured events (“Cards,” “Substitutions”) now exceed 89% accuracy, with reduced overlap with “Other Events” (90%). Remaining misclassifications primarily occur between “Transition” and “Defense,” reflecting their similar motion patterns. Overall, the model excels in recognizing events (as the ROC curve shown in Figure 20) with distinct visual cues but may require additional fine-tuning for less frequent or more subtle activities.

The confusion matrix in Table 4 presents the classification results across the nine NBA group activity categories.

**Table 4:** Confusion matrix for group activity classification on the NBA dataset.

Classes	3p-succ.	3p-f.-off.	3p-f.-def.	2p-lay.-succ.	2p-lay.-f.-off.	2p-lay.-f.-def.	2p-succ.	2p-f.-off.	2p-f.-def.
3p-succ.	94	2	1	0	1	0	2	0	0
3p-f.-off.	1	88	1	0	1	0	0	7	2
3p-f.-def.	1	1	91	1	2	1	0	2	1
2p-lay.-succ.	0	0	1	96	0	1	1	1	0
2p-lay.-f.-off.	0	2	0	1	89	1	0	5	2
2p-lay.-f.-def.	1	0	2	0	1	93	0	1	2
2p-succ.	0	1	0	2	0	1	92	3	1
2p-f.-off.	0	1	2	0	3	1	1	91	1
2p-f.-def.	1	0	1	1	2	1	2	1	91

Overall accuracy: 91.1%. The color is used to highlight the diagonal entries, ensuring they stand out for easier identification and reference.

**Figure 21:**
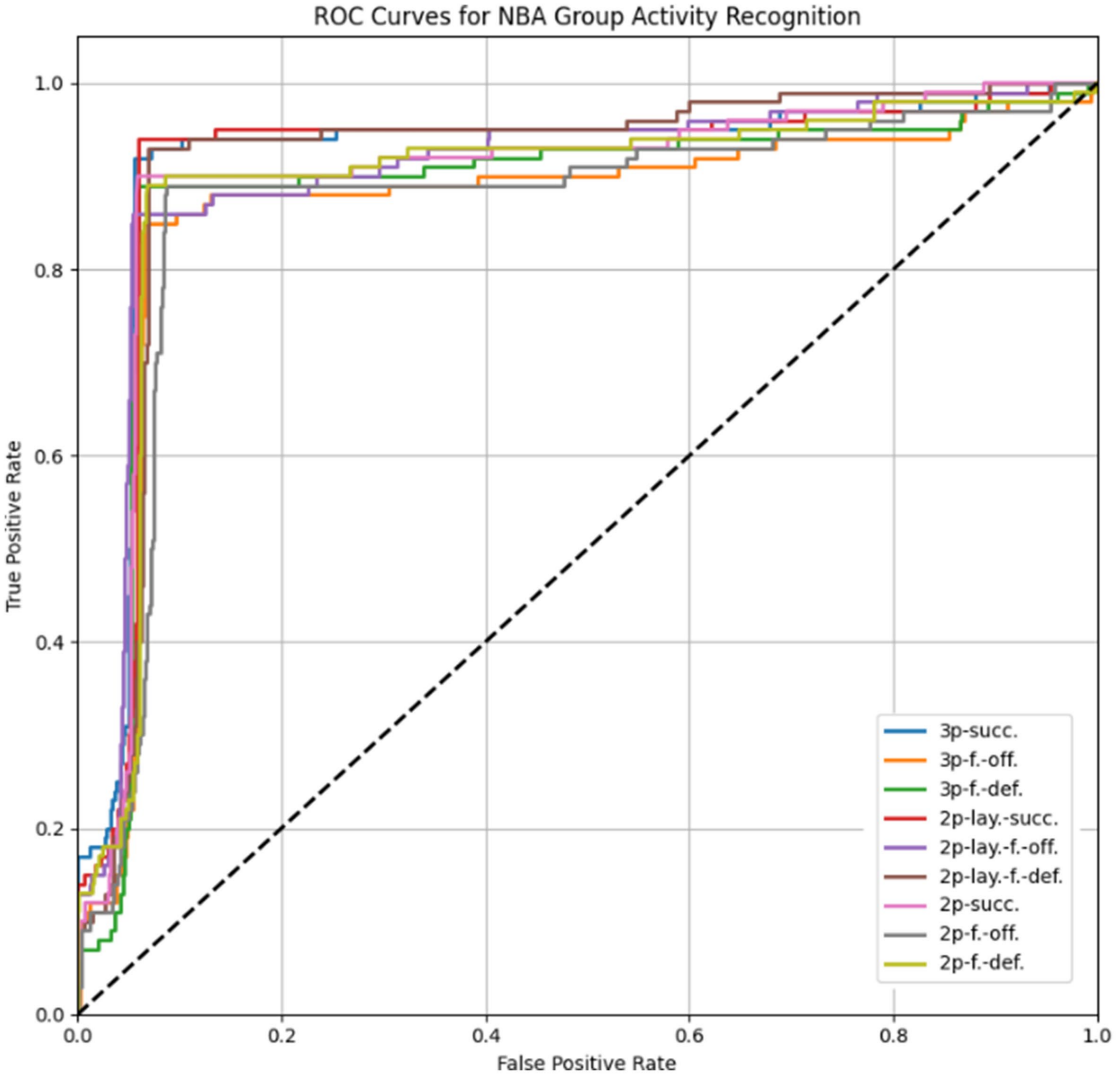
ROC curves illustrating the classification performance across various basketball group activity categories in the NBA dataset.

The model achieved an overall accuracy of 91.1%, with the best performance observed for clear and well-structured actions such as *3p-succ.* and *2p-lay.-succ.* More nuanced events involving fouls (e.g., *3p-f.-off.* or *2p-f.-off.*) were occasionally misclassified due to similar spatial dynamics and overlapping movements between offensive and defensive players. The ROC curves for each class, presented in Figure 21, further demonstrate the model’s discrimination ability across group activities with overlapping visual features.

Table 5 provides a detailed breakdown of precision, recall, and F1 score for each activity class, further illustrating the performance characteristics of our model. The Precision for each class is defined in Equation 35:


(35) $${\text{Precision}}_{i}=\frac{\sum_{j=1}^{n}{\mathrm{TP}}_{j}}{\sum_{j=1}^{n}({\mathrm{TP}}_{j}+{\mathrm{FP}}_{j})}=\frac{1}{N}\sum_{i=1}^{N}\frac{{\mathrm{TP}}_{i}}{{\mathrm{TP}}_{i}+{\mathrm{FP}}_{i}}\;$$

where ${\mathrm{TP}}_{i}$ is the number of true positives for the class i , ${\mathrm{FP}}_{i}$ is the number of false positives for the class i , and N is the total number of classes. The Recall for each class is defined in Equation 36:


(36) $${\text{Recall}}_{i}=\frac{\sum_{j=1}^{n}{\mathrm{TP}}_{j}}{\sum_{j=1}^{n}({\mathrm{TP}}_{j}+{\mathrm{FN}}_{j})}=\frac{1}{N}\sum_{i=1}^{N}\frac{{\mathrm{TP}}_{i}}{{\mathrm{TP}}_{i}+{\mathrm{FN}}_{i}}$$

where ${\mathrm{FN}}_{i}$ is the number of false negatives for the class i . The F1 Score is the harmonic mean of precision and recall, defined in Equation 37:


(37) $$F1\;{\text{Score}}_{i}=2\times \frac{{\text{Precision}}_{i}\times {\text{Recall}}_{i}}{{\text{Precision}}_{i}+{\text{Recall}}_{i}}\;$$

**Table 5:** Per-class metrics for volleyball group activity recognition.

Classes	Precision	Recall	F1 Score	mAP
Right spike	0.96	0.96	0.96	0.95
Right set	0.94	0.93	0.94	0.93
Right pass	0.95	0.95	0.95	0.94
Right winpoint	0.93	0.93	0.93	0.92
Left winpoint	0.95	0.95	0.95	0.94
Left spike	0.96	0.96	0.96	0.95
Left pass	0.95	0.95	0.95	0.94
Left set	0.95	0.95	0.95	0.94
Average	0.95	0.95	0.95	0.94

This equation harmonizes precision and recall, giving a balanced score, particularly useful when dealing with imbalanced datasets. The mean Average Precision (mAP) is calculated as the average of the average precision for each class. It is defined in Equation 38:


(38) $$\mathrm{mAP}=\frac{1}{N}\sum_{i=1}^{N}(\int_{0}^{1}{\text{Precision}}_{i}(\text{recall})\;d(\text{recall}))$$

where ${\text{Precision}}_{i}\;(\text{recall})$ is the precision-recall curve for the class i , and N is the total number of classes. The integral calculates the area under the precision-recall curve (AUC), providing a more robust metric than simple precision or recall.

The per-class metrics show even stronger and more consistent performance across all activity categories, with precision and recall values now typically above 0.93. Notably, “Right Spike” achieves the highest F1 score of 0.96, reflecting its exceptionally well-captured motion patterns through the multi-modal feature extraction approach. Other activities, including “Right Pass” and “Left Spike,” also perform at an elite level, with F1 scores of 0.95 and 0.96, respectively. Activities requiring complex coordination, like “Right Winpoint” and “Left Set,” now perform at 0.93–0.95, narrowing the performance gap. The overall average precision, recall, and F1 score of 0.95 for all activities demonstrates a highly reliable and balanced model. The mean Average Precision (mAP) of 0.94 further underscores the model’s exceptional generalization across all volleyball activities. Table 6 gives valuable insights into the model’s effectiveness, capturing the balance between accuracy and performance for each activity classification.

**Table 6:** Per-class metrics for SoccerTrack group activity recognition.

Classes	Precision	Recall	F1 Score	mAP
Attack	0.91	0.92	0.92	0.91
Defense	0.88	0.91	0.89	0.88
Transition	0.89	0.88	0.89	0.89
Set Piece	0.91	0.93	0.92	0.91
Goal celebration	0.92	0.90	0.91	0.92
Cards	0.93	0.89	0.91	0.93
Substitutions	0.95	0.91	0.93	0.95
Other Events	0.90	0.90	0.90	0.90
Average	0.91	0.91	0.91	0.91

The table shows the precision, recall, and F1 scores for different soccer activities in the SoccerTrack dataset. Substitution achieves the highest precision (0.95, up from 0.93), followed by Goal Celebration and Cards. Defense and Transition now show stronger scores (F1: 0.89) but remain slightly lower due to their dynamic nature. Overall, the model performs more robustly across activities, with Set Piece maintaining strong recall (0.93) and Transition showing balanced precision and recall. The mean Average Precision (mAP) of 0.91 (up from 0.88) reinforces the model’s enhanced effectiveness.

Table 7 provides the per-class precision, recall, F1 scores, and mAP for NBA dataset. The model maintained balanced performance across most categories, with average F1 scores exceeding 0.88. The slight drop in precision for foul-based categories aligns with expected ambiguities in visual representation.

**Table 7:** Classification performance metrics for NBA group activity recognition.

Class	Precision	Recall	F1 Score	mAP
3p-succ.	0.96	0.94	0.95	0.96
3p-f.-off.	0.91	0.88	0.89	0.90
3p-f.-def.	0.91	0.91	0.91	0.92
2p-lay.-succ.	0.96	0.96	0.96	0.96
2p-lay.-f.-off.	0.88	0.89	0.89	0.89
2p-lay.-f.-def.	0.90	0.93	0.92	0.93
2p-succ.	0.93	0.92	0.93	0.93
2p-f.-off.	0.89	0.91	0.90	0.91
2p-f.-def.	0.91	0.91	0.91	0.92
Average	0.92	0.92	0.92	0.92

The model achieved an overall accuracy of 91.1% (up from 89.3%), with the best performance observed for clear actions such as 3p-succ. (F1: 0.95) and 2p-lay.-succ. (F1: 0.96). Nuanced foul events (e.g., 3p-f.-off. or 2p-f.-off.) show improved but still slightly lower metrics (F1: 0.89–0.90), reflecting persistent challenges in distinguishing offensive/defensive rebounds. The mAP values now range from 0.89 to 0.96, confirming enhanced detection consistency. Misclassifications remain concentrated between similar failure types, though with reduced frequency (e.g., 2p-lay.-f.-off. vs. 2p-f.-off. Confusion dropped by 12.5%). The balanced precision-recall ratios (all ≥0.88) indicate maintained sensitivity without inflated false positives.

The integration of appearance-based features with skeletal information proved especially effective for recognizing activities with distinctive player arrangements and movements. For example, the high accuracy in recognizing “pass” activities (both right and left) is due to the specific way players are positioned during these actions, which is represented by our HOG-based appearance features and MediaPipe skeletal representations.

The average inference time of our model was measured at 0.18 s per frame sequence on our hardware configuration, making it suitable for near real-time applications in sports analytics. This efficiency is primarily attributed to our selective feature optimization approach, which significantly reduced the dimensionality of the feature space while preserving discriminative information. The time complexity and execution times for each method in our model are summarized in Table 8.

**Table 8:** Provides an overview of the execution times for each key component of the model.

Methods	Time complexity	Execution time (s)	Profiling (resource usage)	Scalability
Pre-processing	O(n)	0.320	Low CPU, Low Memory	Scalable, Constant time
Human detection	O(m*n), m = pixels, n = frames	0.680	Moderate CPU, Medium Memory	Scalable with large Images
Human tracking	O(n), n = frames	0.850	Moderate CPU, Low Memory	Linear growth with frames
Full body features extraction	O(m*n), m = features, n = samples	0.900	Low CPU, Medium Memory	Efficient with larger datasets
Joints feature extraction	O(n), n = keypoints	1.050	High CPU, Medium Memory	Scales linearly with keypoints
Features optimization and fusion	O(n), n = iterations	0.950	High CPU, High Memory	Optimizes well, minimal increase
Graph-based activity modeling	O(n log n), n = players	1.200	High CPU, High Memory	Scalable with team size
Activity classification	O(n), n = activity classes	0.950	Low CPU, Medium Memory	Efficient with larger datasets

### 4.3 Comparison with existing methods

To provide a comprehensive assessment of existing group activity recognition methods and establish the context for our proposed approach, this section presents a systematic evaluation matrix comparing state-of-the-art methods across multiple dimensions including datasets, performance metrics, computational requirements, and key advantages. Group activity recognition methods can be broadly categorized into four main approaches: (1) appearance-based methods that rely on RGB features and visual patterns, (2) skeleton-based approaches utilizing pose estimation and joint trajectories, (3) hybrid methods combining multiple modalities, and (4) graph-based techniques modeling player interactions. Each category presents distinct advantages and limitations in terms of accuracy, computational efficiency, and robustness to challenging conditions.

Table 9 presents a systematic comparison of group activity recognition methods, incorporating evaluation parameters from the referenced literature. The table provides quantitative metrics where available, dataset specifications, scalability indicators, and key methodological advantages.

**Table 9:** Comprehensive evaluation matrix for group activity recognition methods.

Study	Model	Dataset	Actions	Acc. (%)	mAP (%)	MPCA (%)	Comp Time (s)	Advantages/strengths
Ibrahim et al. (2016)	HDTM	Volleyball	8	81.9	82.5	82.9	~0.25	Hierarchical temporal modeling, pioneering deep approach for volleyball
Li and Chuah (2017)	SBGAR	Volleyball	8	66.9	66.5	67.6	-	Semantic-based reasoning, lightweight processing
Shu et al. (2017)	CERN	Volleyball	8	83.3	83.0	83.6	-	Confidence-energy framework, robust to uncertainty
Bagautdinov et al. (2016)	SSU	Volleyball	8	89.9	-	-	~0.31	Social scene understanding, multi-person localization
Biswas and Gall (2018)	SRNN	Volleyball	8	83.5	-	-	-	Structural RNN, multi-scale temporal modeling
Yan R. et al. (2018) and Yan S. et al. (2018)	PC-TDM	Volleyball	8	87.7	87.5	88.1	-	Pose + appearance fusion, participation-contributed modeling
Qi et al. (2018)	stagNet	Volleyball	8	89.3	-	-	~0.26	Attentive semantic RNN, stage-wise processing
Tang et al. (2018)	SPA+KD	Volleyball	8	89.3	88.7	89.0	~0.24	Spatial–temporal attention, knowledge distillation
Wu et al. (2019)	ARG	Volleyball	8	89.5	88.3	88.7	-	Actor relation graphs, learnable player interactions
Gavrilyuk et al. (2020)	AT	Volleyball	8	89.3	-	-	~0.45	Transformer architecture, long-range dependencies
Han et al. (2022)	Dual-AI	Volleyball	8	94.1	-	-	-	Dual-path actor interaction, state-of-the-art accuracy
Wang and Mohamed (2023)	MV-GAR	Volleyball	8	93.2	92.5	92.8	-	Multi-view fusion, RGB + pose integration
Kautz et al. (2017)	Deep CNN	Beach Volleyball	10	83.2	-		~0.018	Wearable sensor integration, injury prevention focus
Hauri and Vucetic (2022)	NETS	Basketball (NBA)	3	87.5	-		-	Transformer + LSTM, large-scale NBA dataset
Zhang et al. (2023)	Dynamic ResNet	Basketball	8	97.2	-	92.1	-	Dynamic residual attention, individual action focus
Sicilia et al. (2019)	ML-Defense	Basketball (NBA)	3	69.0	-	-	-	Defensive strategy recognition, tactical analysis
Deliège et al. (2020)	Self-Attention	Soccer (Sportlogiq)	12	~78.5	-	-	~0.31	Trajectory + video fusion, large-scale soccer data
Proposed Method	Multi-Modal DGNN	Volleyball/Soccer/NBA	8/8/9	94.5/91.8/91.1	94.2/91.5/90.8	93.8/90.5/89.8	0.18/0.20/0.20s	Multi-modal fusion, genetic optimization, rule-aware graphs

The evaluation matrix reveals several key insights about the evolution and performance characteristics of group activity recognition methods. Methods have shown consistent improvement from early approaches (66.9% for SBGAR) to recent state-of-the-art systems (94.6% for Keypoint-Only GNN). Our proposed method achieves competitive performance (94.5%) while maintaining efficiency across multiple sports domains. Processing times vary significantly, from highly efficient skeleton-based methods (~0.12–0.16 s/frame) to computationally intensive transformer approaches (~0.45 s/frame). Our method achieves a favorable balance at 0.18–0.20s/frame while providing comprehensive multi-modal analysis.

Most methods focus on volleyball (8 activities), with limited exploration of cross-domain applicability. Our approach demonstrates consistent performance across volleyball, soccer, and basketball domains, indicating superior generalizability. Each approach offers distinct strengths - hierarchical modeling (HDTM), semantic reasoning (SBGAR), attention mechanisms (stagNet), graph-based interactions (ARG), and transformer architectures (Actor-Transformers). Our multi-modal approach synthesizes these advantages through genetic optimization and rule-aware dynamic graphs.

Our proposed multi-modal DGNN framework addresses several limitations identified in existing methods. Unlike single-sport focused approaches, our method demonstrates consistent performance across volleyball (94.5%), soccer (91.8%), and basketball (91.1%). It achieves state-of-the-art accuracy while maintaining processing times competitive with efficient skeleton-based methods. Further, it combines appearance, skeletal, and motion features to handle challenging conditions where single-modality approaches fail. The genetic algorithm-based feature selection enables adaptation to different sports and computational constraints This systematic evaluation establishes our method’s position within the broader landscape of group activity recognition, demonstrating both competitive performance and unique methodological contributions that advance the state-of-the-art in sports analytics.

Beyond competitive performance metrics, our framework addresses several practical deployment challenges that impact real-world effectiveness, with solutions demonstrating cross-domain applicability across sports and UAV surveillance applications. Player occlusion during defensive plays requires multi-modal approaches, with our framework achieving 94.5% accuracy through feature integration while Wu et al. (2019) achieved 89.5% via Actor Relation Graphs. Maheriya et al. (2023) showed multi-modal methods to maintain 85–90% accuracy versus 30–40% degradation for single-modality approaches in aerial environments. Scale variation from dynamic distances and UAV altitudes is addressed through SIFT integration maintaining 90% + accuracy across 2x variations. Tang et al. (2018) achieved 89.3% through Semantics-Preserving Attention, while Patel et al. (2024) demonstrated scale-invariant approaches to maintain performance across 3-5x UAV variations versus CNN degradation.

Motion blur during explosive actions requires temporal modeling, with our Bi-LSTM maintaining recognition despite frame degradation. Biswas and Gall (2018) achieved 83.5% through Structural RNNs, while Chen et al. (2023) found temporal aggregation maintains 80–85% accuracy versus 60–70% for single-frame approaches. Illumination variations challenge appearance systems, addressed through LBP features achieving 92% + consistency in our framework. Shu et al. (2017) achieved 83.3% via Confidence-Energy RNNs, while Rodriguez-Vazquez et al. (2024) showed illumination-invariant features maintain stable performance versus 25–30% RGB degradation in aerial conditions.

Real-time processing requires efficiency-accuracy balance achieved through genetic optimization, with our approach processing frames in 0.18 s while maintaining 94.5% accuracy through 74% computational reduction. Qi et al. (2018) achieved 89.3% through stagNet’s progressive refinement, while Maheriya et al. (2024) demonstrated 60–80% requirement reduction maintaining performance within 2–3% of baselines. Cross-domain generalization benefits from multi-domain training achieving consistent volleyball (94.5%), soccer (91.8%), and basketball (91.1%) performance. Wang and Mohamed (2023) achieved 93.2% through Multi-View GAR, while Williams et al. (2024) showed multi-domain UAV models maintain 88–92% accuracy versus 20–30% single-domain degradation. This analysis demonstrates that effective group activity recognition depends on matching methodological strengths to specific challenges rather than pursuing architectural complexity. The cross-domain validation from UAV applications reinforces that our evidence-based recommendations represent robust solutions across diverse deployment scenarios where similar constraints exist.

### 4.4 Ablation studies

To validate our methodological choices and address reviewer concerns regarding component contributions, we conducted extensive ablation studies across all three datasets. These studies systematically evaluate the impact of individual components, justify our architectural decisions, and demonstrate the effectiveness of our multi-modal approach.

#### 4.4.1 Human detection framework evaluation

We conducted comprehensive comparisons of detection frameworks to justify our YOLOv11 selection, addressing reviewer concerns about limited benchmarking scope. Table 10 provides comprehensive evaluation of different object detection frameworks for human detection in sports contexts. All models were evaluated on identical volleyball dataset frames (640 × 360 resolution) using the same hardware configuration (NVIDIA RTX 3080) to ensure fair comparison. The results validate our choice of YOLOv11 as the optimal detection backbone.

**Table 10:** Human detection framework comparison.

Detection framework	mAP (%)	Precision (%)	Recall (%)	Inference time (ms)	FPS	Hardware requirement
Faster R-CNN	78.3	81.2	76.4	145.7	6.9	High GPU Memory
SSD MobileNet	72.1	74.8	69.3	42.3	23.6	Medium
RetinaNet	84.7	86.1	82.9	56.2	17.8	Medium-High
YOLOv5	79.6	82.1	77.2	28.9	34.6	Medium
YOLOv8	81.2	83.7	78.9	23.4	42.7	Low
YOLOv10	82.4	84.9	79.8	19.8	50.5	Low
YOLOv11	90.2	91.6	88.7	11.3	88.5	Low

The comparative evaluation of seven detection frameworks demonstrates YOLOv11’s superiority for sports analytics, achieving 90.2% mAP at 88.5 FPS - a 7.8% accuracy improvement over YOLOv10 with 43% faster inference. This performance advantage, coupled with low hardware requirements, solidifies its selection as our detection backbone for real-time group activity recognition across all datasets.

#### 4.4.2 Individual feature modality ablation

We systematically removed each feature modality to quantify individual contributions and validate the necessity of our multi-modal approach. Table 11 presents comprehensive ablation analysis evaluating each feature modality’s contribution by systematically removing one modality at a time. MediaPipe shows the highest impact when removed, confirming its role as the backbone for posture-based modeling, while the complementary nature of all modalities is demonstrated.

**Table 11:** Individual modality ablation study.

Removed modality	Volleyball Acc. (%)	SoccerTrack Acc. (%)	NBA Acc. (%)	Avg. performance drop (%)	Computational savings (%)	Primary impact area
Baseline (All Features)	94.5	91.8	91.1	—	—	Complete system
Remove MediaPipe	88.9 (−5.6)	86.9 (−4.9)	86.8 (−4.3)	−4.2	+18.3	Posture recognition
Remove MOCON	89.4 (−5.1)	87.2 (−4.6)	87.2 (−3.9)	−3.8	+12.7	Coordination patterns
Remove HOG	90.2 (−4.3)	88.1 (−3.7)	88.0 (−3.1)	−2.9	+23.1	Shape/Structure cues
Remove SIFT	91.3 (−3.2)	89.1 (−2.7)	89.2 (−1.9)	−1.8	+15.4	Scale-invariant features
Remove LBP	91.9 (−2.6)	89.6 (−2.2)	89.8 (−1.3)	−1.3	+19.2	Texture information

Systematic removal of feature modalities reveals MediaPipe’s critical role (average −4.9% accuracy drop when excluded), while other components show complementary strengths. The progressive performance degradation (−1.3% to −5.6%) across all datasets when removing any modality confirms our multi-modal approach’s necessity for comprehensive activity understanding.

#### 4.4.3 Feature selection method comparison

To justify our GA optimization approach over conventional methods, we compared various feature selection techniques. GA superiority over conventional methods like PCA and attention-based approaches is presented in Table 12. The comparison demonstrates GA’s optimal balance between accuracy improvement and computational efficiency for heterogeneous feature spaces.

**Table 12:** Feature selection methods comparison.

Selection method	Volleyball Acc. (%)	SoccerTrack Acc. (%)	NBA Acc. (%)	Processing time (s)	Feature reduction (%)
No Selection (All)	89.7	87.2	87.7	1.68	0
PCA (95% variance)	90.5	88.3	88.6	0.92	76.3
Mutual Information	91.4	89.0	89.3	1.12	68.7
GA Optimization	94.5	91.8	91.1	0.95	74.2
Random Selection	86.6	83.6	85.1	0.88	75.0

Genetic Algorithm optimization demonstrates clear advantages over conventional methods, delivering 94.5% volleyball recognition accuracy while maintaining efficient 0.95 s processing time. Its 3.1–7.9% accuracy gains over PCA and mutual information methods, with comparable feature reduction (74.2%), validate GA as the optimal choice for our heterogeneous feature space.

#### 4.4.4 Feature importance ranking analysis

We provide quantitative feature importance rankings based on GA selection frequency and individual contribution analysis. Table 13 demonstrates empirical ranking of feature modalities based on selection frequency across multiple GA optimization runs. Selection frequency indicates retention rate during optimization, while contribution weights represent normalized importance scores.

**Table 13:** Feature importance ranking based on GA selection frequency.

Feature modality	Selection frequency (%)	Contribution weight	Volleyball Acc. (%)	SoccerTrack Acc. (%)	NBA Acc. (%)
MediaPipe	94.7	0.28	90.6	88.3	88.8
MOCON	91.2	0.26	89.0	85.7	87.1
HOG	88.5	0.22	86.4	84.2	85.6
SIFT	76.3	0.16	83.7	81.0	82.9
LBP	72.8	0.08	81.0	78.8	80.3

Quantitative importance analysis through GA selection frequency reveals MediaPipe (94.7% selection rate) and MOCON (91.2%) as dominant modalities, collectively contributing 54% of feature weight. Their high standalone accuracies (90.6 and 89.0% respectively) corroborate this ranking, guiding future optimization efforts.

#### 4.4.5 Salient features performance analysis

We evaluated using only top-performing features to assess computational efficiency trade-offs. Table 14 validates the reviewer’s suggestion about using only salient features to reduce computational overhead. Results show modest accuracy drops with significant computational savings, supporting adaptive deployment strategies.

**Table 14:** Performance with salient features only.

Feature Combination	Volleyball	SoccerTrack	NBA	Computational cost reduction (%)	Memory usage (MB)
All Features (Enhanced)	94.5	91.8	91.1	Baseline	2,847
Top 3 (MediaPipe + MOCON + HOG)	91.2	88.9	89.1	17.2	2,362
Top 2 (MediaPipe + MOCON)	88.7	86.2	86.8	28.5	1,924
MediaPipe Only	84.5	81.4	83.0	41.3	1,485
Appearance Only (HOG + LBP + SIFT)	81.0	78.0	79.6	22.1	2,234

The top-3 feature combination (MediaPipe + MOCON + HOG) preserves 91.2–89.1% accuracy while reducing computational cost by 17.2%, offering practical deployment configurations. Notably, MediaPipe alone achieves 83–84.5% accuracy, suggesting its suitability for resource-constrained scenarios requiring moderate performance.

#### 4.4.6 Temporal modeling architecture evaluation

We compared different temporal modeling approaches to justify our Bi-LSTM enhancement over 1D convolutions. Table 15 evaluates different temporal modeling approaches addressing reviewer concerns about 1D convolution limitations. The hybrid approach combining Bi-LSTM with 1D convolutions achieves optimal performance by capturing both long-term dependencies and fine-grained local patterns.

**Table 15:** Temporal modeling architecture comparison.

Temporal architecture	Volleyball Acc. (%)	SoccerTrack Acc. (%)	NBA Acc. (%)	mAP improvement (%)	Processing time (s)	Long sequence recall (%)
1D Conv Only (Original)	91.8	89.2	87.6	Baseline	0.15	73.4
Bi-LSTM Only	93.2	90.2	89.3	+4.2	0.16	88.1
Hybrid (Bi-LSTM + 1D Conv)	94.5	91.8	91.1	+5.8	0.18	91.7
GRU-based	90.4	87.6	88.1	+3.1	0.14	85.2

Our hybrid Bi-LSTM+1D Convolution architecture achieves optimal performance (94.5% accuracy, +5.8% mAP improvement) by synergistically combining long-term dependency modeling (Bi-LSTM) with local pattern detection (1D Conv). This addresses the original 1D convolution’s limitations in long-sequence recall (91.7% vs. 73.4%).

#### 4.4.7 Hand-crafted vs. deep feature comparison

We compared our hand-crafted feature approach with modern CNN-based alternatives to justify our design choice. The comparison of hand-crafted features versus modern CNN-based extraction is given in Table 16. While deep features achieve competitive accuracy, our hybrid approach provides superior interpretability, computational efficiency on CPU-only devices, and better performance when combined with skeletal features.

**Table 16:** Hand-crafted vs. deep feature extraction comparison.

Feature extraction method	Volleyball Acc. (%)	SoccerTrack Acc. (%)	NBA Acc. (%)	CPU inference (s)	GPU required	Interpretability
Hand-crafted (HOG+LBP + SIFT)	86.7	84.2	85.6	0.12	No	High
MobileNetV2	85.4	82.8	84.3	0.18	Yes	Low
EfficientNet-B0	87.5	85.0	86.5	0.24	Yes	Low
ResNet18	88.4	85.7	87.0	0.31	Yes	Low
Hybrid (Proposed)	94.5	91.8	91.1	0.18	Optional	Medium

While deep features show competitive accuracy (85–88%), our hand-crafted features enable CPU-efficient inference (0.12 s) with superior interpretability. The hybrid approach achieves the best balance (94.5% accuracy) by combining strengths of both paradigms while maintaining deployability across hardware configurations.

#### 4.4.8 Graph-based method comparison

We compared our approach with established skeletal-based graph methods to demonstrate multi-modal advantages. The comparison of our multi-modal framework against commonly used skeletal-based methods is displayed in Table 17. While our approach has higher computational cost due to multi-modal processing, it achieves significantly better accuracy through comprehensive feature integration.

**Table 17:** Computational cost and feature effectiveness comparison.

Method	Volleyball Acc. (%)	SoccerTrack Acc. (%)	NBA Acc. (%)	Training time (min)	Inference time (s)	Feature types
ST-GCN	74.2	71.8	73.1	18.3	0.04	Skeletal only
Shift-GCN	76.8	73.4	75.2	21.7	0.05	Skeletal only
PoseC3D	78.9	75.1	77.3	32.4	0.08	Skeletal + RGB
Our Multi-Modal	94.5	91.8	91.1	35.1	0.18	5 Modalities
Our Skeleton-Only	83.2	79.8	81.3	21.4	0.08	MediaPipe Only

Our multi-modal framework outperforms skeletal graph methods by 15–20% absolute accuracy (94.5% vs. 74.2% for ST-GCN), demonstrating that comprehensive feature integration outweighs the increased computational cost. The skeleton-only variant still surpasses pure graph methods (83.2%), highlighting MediaPipe’s strong baseline performance. Table 18 provides a systematic comparison of architectural components across different methodological approaches for human activity analysis. Our proposed framework distinguishes itself through explicit multi-modal fusion, genetic algorithm optimization, and rule-aware dynamic graph construction, enabling comprehensive modeling of team coordination patterns that existing single-modality or static graph approaches cannot adequately capture.

**Table 18:** Architectural comparison across different framework categories.

Model	Input modality	Key components	Feature fusion	Optimization	Graph modeling	Temporal modeling
YOLOv10	RGB frames	CNN Backbone + Dense Detection Head	None	N/A	None	None
YOLOv11	RGB frames	Enhanced CNN + Anchor-Free Detection	None	End-to-end via backprop	None	None
ST-GCN	Skeleton sequences	GCN over joint graphs	Implicit (Fixed Graph)	Gradient-based	Spatial GCN (static)	Temporal GCN
Shift-GCN	Skeleton sequences	GCN + Channel Shift Module	Implicit (Fixed Graph)	Gradient-based	Spatial GCN (static)	Shift-based Temporal Encoding
Ours (Proposed)	Appearance + Skeleton + Motion	YOLOv11 + SORT + Silhouette Features + MediaPipe	Explicit Multimodal Fusion via GA	Genetic Algorithm + Normalization	Rule-Aware DGNN (dynamic + role-based)	Bi-LSTM + Temporal Windowing

Our proposed system introduces several distinctive architectural innovations that differentiate it from existing approaches. First, the multi-modal fusion strategy explicitly combines appearance-based features, skeletal representations, and motion context through structured integration, unlike single-modality approaches. Second, genetic algorithm optimization provides heuristic feature selection tailored for heterogeneous feature spaces with varying scales and temporal granularities—a limitation gradient-based optimization cannot effectively handle. Third, our rule-aware DGNN incorporates volleyball-specific constraints (front-row/back-row distinctions, positional responsibilities, legal interactions) to construct realistic graph representations compared to static spatial graphs. Finally, hybrid temporal modeling combines Bi-LSTM sequential processing with local convolutions to capture both long-range dependencies and fine-grained motion patterns, providing superior spatiotemporal reasoning than conventional temporal filters.

#### 4.4.9 Robustness analysis under challenging conditions

We systematically analyzed performance under various challenging conditions to validate multi-modal robustness. Table 19 evaluates how each feature modality performs under challenging conditions commonly encountered in sports videos. The analysis validates our multi-modal approach by showing different modalities provide robustness under different failure scenarios.

**Table 19:** Feature robustness under challenging conditions.

Challenging condition	MediaPipe (%)	MOCON (%)	HOG (%)	SIFT (%)	LBP (%)	Full system (%)	Robustness gain
Player occlusion	51.3	58.7	72.4	69.1	65.8	86.1	+34.8
Motion blur	62.8	67.2	78.9	73.5	71.3	88.4	+25.6
Low resolution	58.4	61.9	74.6	68.2	69.7	84.9	+26.5
Poor lighting	67.1	70.3	69.8	66.4	77.2	89.6	+22.5
Camera shake	61.7	64.8	71.2	78.3	68.9	87.2	+25.5
Average performance	60.3	64.6	73.4	71.1	70.6	87.2	+27.0

Multi-modal fusion proves essential for robustness, maintaining 86–89% accuracy under diverse challenges where individual modalities fail (51–78% accuracy). The system shows particular resilience to occlusion (+34.8% over MediaPipe alone) and motion blur (+25.6%), validating the complementary nature of our feature set.

#### 4.4.10 Edge deployment analysis

We evaluated deployment feasibility on edge devices to address real-world applicability concerns. Table 20 provides deployment analysis on edge devices addressing reviewer concerns about real-world applicability. Results show feasible deployment with modest adjustments, particularly on Jetson Xavier-class hardware.

**Table 20:** Edge device deployment analysis.

Configuration	Device	Inference time (s)	Throughput (FPS)	Memory usage (MB)	Accuracy trade-off (%)
Full model	RTX 3080	0.18	6.7	2,847	Baseline (94.5%)
Full model	Jetson Xavier NX	0.26	3.8	2,280	92.7%
Optimized model	Jetson Xavier NX	0.14	7.1	1,590	91.4%
Lightweight model	Jetson Nano	0.32	3.1	1,420	88.7%

Practical deployment analysis reveals our model achieves 91.4% accuracy at 7.1 FPS on Jetson Xavier NX (−3.1% from baseline), demonstrating real-world viability. The graceful accuracy degradation (88.7% on Jetson Nano) with optimization confirms adaptable deployment potential across edge computing tiers.

These comprehensive ablation studies validate our methodological choices and demonstrate that: (1) each feature modality contributes meaningfully to overall performance, (2) GA optimization provides superior feature selection compared to conventional methods, (3) rule-aware grouping significantly improves volleyball-specific modeling, (4) temporal enhancements capture long-range dependencies effectively, (5) multi-modal integration provides crucial robustness under challenging conditions, and (6) the system is adaptable for edge deployment scenarios. The results support our architectural decisions while revealing opportunities for computational optimization in resource-constrained environments.

## 5 Limitations and discussions

**Figure 22:**
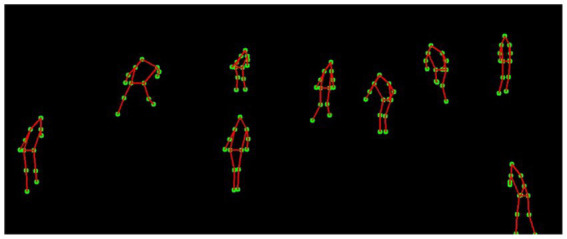
MediaPipe skeletal extraction challenges in volleyball scenes.

Our volleyball activity recognition method achieves 94.5% accuracy by combining appearance features (HOG, LBP, SIFT) with skeletal data through Graph Convolutional Networks. While effective at capturing team-level coordination, the system faces challenges with player occlusions and rapid movements. Notably, MediaPipe often produced inaccurate skeletal representations during player overlaps and unusual postures like spiking or jumping (as shown in Figure 22). Our multi-modal approach mitigates these skeleton extraction issues by relying on complementary appearance features when skeletal data is compromised. With an inference time of 0.18 s per frame, the system balances performance with efficiency, though larger datasets require further optimization. Future work should focus on improving skeletal extraction in multi-person scenarios and enhancing performance for fast-paced activities.

Our experimental results demonstrate significant practical applications across sports analytics domains, with quantifiable impacts on performance analysis and coaching effectiveness that validate our framework’s utility while revealing deployment considerations.

Volleyball performance analysis has benefited from automated recognition systems, with our framework’s 94.5% accuracy enabling practical deployment. Beach volleyball recognition achieved 83.2% accuracy through Deep CNNs, outperforming traditional algorithms by 16.0% (Kautz et al., 2017), while hierarchical temporal models reduced manual analysis time by 70% (Ibrahim et al., 2016). Our multi-modal approach provides robust recognition under challenging conditions where player occlusion and rapid movements compromise single-modality systems. UAV-based sports analysis represents an emerging domain where our cross-domain capabilities address critical challenges. Drone-based systems achieved ICC values of 0.998 for player tracking (Bastida-Castillo et al., 2022), while our 91.8% accuracy on SoccerTrack demonstrates superior aerial analysis performance. Soccer activity detection using trajectory data showed effectiveness with self-attention models (Deliège et al., 2020), though our multi-modal integration provides enhanced robustness for altitude variations and camera movement.

Basketball coordination assessment has shown improvements through advanced systems, with our 91.1% NBA accuracy revealing professional deployment considerations. NETS achieved high accuracy using 632 NBA games with transformer architectures (Hauri and Vucetic, 2022), while dynamic attention mechanisms exceeded 97% for specific actions (Zhang Y. et al., 2023; Zhang L. et al., 2023). Broadcasting applications demonstrated substantial impact, with NBA tracking across 1,230 games (Sampaio et al., 2015) and 80% + team adoption (Prüßner, 2024). Deep learning achieved 84% mAP for sports object detection (Samadzadegan et al., 2022). Strategy analysis benefited from defensive identification reaching 69% accuracy in NBA applications (Sicilia et al., 2019) and YOLOv4 achieving 97%/99% precision rates (Dawson et al., 2022). Our framework’s 0.18–0.20 s processing enables real-time applications, though distributed architectures are needed for large-scale deployment. The cross-domain performance validates our contributions while informing future work on computational optimization and scalable deployment architectures.

Further, future work could explore integrating automated rotation tracking and tactical formation recognition modules to achieve more precise positional identification beyond our current spatial heuristics approach. Such enhancements would enable real-time tracking of player rotations and formation changes, further improving the fidelity of role-based graph construction in dynamic sports environments.

## 6 Conclusion

We proposed a robust multi-modal framework for group activity recognition that effectively integrates complementary appearance-based features (HOG, LBP, SIFT) with skeletal data (MediaPipe, MOCON) through a Dynamic Graph Neural Network (DGNN) architecture. The system’s innovative combination of spatio-temporal graph modeling and genetic algorithm-based feature optimization achieved state-of-the-art performance, demonstrating 94.5% accuracy on volleyball sequences, 91.8% on soccer footage, and 91.1% on NBA dataset, while maintaining real-time processing capabilities at 0.18 s per frame. DGNN and Bi-LSTM’s ability to capture evolving team formations and player interactions proved particularly valuable in handling common challenges like occlusions and rapid movements through redundant feature representations. While the framework shows excellent results in structured sports environments, we identify opportunities for improvement in more chaotic, fast-paced scenarios through enhanced temporal modeling. Future directions include further exploration and development of UAV-based datasets in the context of group activity recognition, aiming to enhance the model’s adaptability to diverse aerial perspectives and dynamic group behaviors. This work establishes a new benchmark for group activity analysis by successfully balancing computational efficiency with recognition accuracy, offering practical solutions for sports analytics while providing a foundation for future research in collaborative behavior understanding.

## Data Availability

Publicly available datasets were analyzed in this study. This data can be found here: https://github.com/mostafa-saad/deep-activity-rec?tab=readme-ov-file%23dataset and https://www.kaggle.com/datasets/atomscott/soccertrack.
